# GWAS Identifies SNP Markers and Candidate Genes for Off-Flavours and Protein Content in Faba Bean (*Vicia faba* L.)

**DOI:** 10.3390/plants14020193

**Published:** 2025-01-11

**Authors:** Antonio Lippolis, Boudewijn Hollebrands, Valentina Acierno, Catrienus de Jong, Laurice Pouvreau, João Paulo, Salvador A. Gezan, Luisa M. Trindade

**Affiliations:** 1 Plant Breeding, Wageningen University & Research, Droevendaalsesteeg 1, 6708 PB Wageningen, The Netherlands; antonio.lippolis@wur.nl; 2 Unilever Foods Innovation Centre—Hive, Bronland 14, 6708 WH Wageningen, The Netherlands; boudewijn.hollebrands@unilever.com; 3 Laboratory of Organic Chemistry, Wageningen University & Research, Stippeneng 4, 6708 WE Wageningen, The Netherlands; 4 Wageningen Food & Biobased Research, Wageningen University & Research, Bornse Weilanden 9, 6708 WG Wageningen, The Netherlands; valentina.acierno@wur.nl (V.A.); catrienus.dejong@wur.nl (C.d.J.); laurice.pouvreau@wur.nl (L.P.); 5 Biometris, Wageningen University & Research, Droevendaalsesteeg 1, 6708 PB Wageningen, The Netherlands; joao.paulo@wur.nl; 6 VSN International Ltd., Hemel Hempstead HP2 4TP, UK; salvador.gezan@vsni.co.uk

**Keywords:** faba bean, GWAS, genetic diversity, off-flavour, plant-based foods, lipid-oxidation, hexanal, tannins, protein

## Abstract

Faba bean (*Vicia faba* L.) is a valuable ingredient in plant-based foods such as meat and dairy analogues. However, its typical taste and aroma are considered off-flavours in these food applications, representing a bottleneck during processing. Breeding is needed to develop varieties with minimal off-flavours and high protein content. The genetic regulation of these traits is underexplored. To dissect their genetic architecture, we performed a genome-wide association study (GWAS). A total of 245 faba bean accessions (the CGN population) were genotyped using the 90K-SPET targeted assay. These accessions were phenotyped in 2021 and 2022 in the Netherlands for protein, oil, fatty acids, lipid-derived products, phenolic acids, flavonoids, and tannins. The CGN population showed large phenotypic variation and moderate-to-high narrow-sense heritability for most traits. The growing environment significantly affected all traits, with trait-specific genotype-by-year (GxY) interactions. Condensed tannins and fatty acids were the most stable across the two years and had the highest heritability estimates (*h*^2^ > 0.6). GWAS identified a total of 148 single nucleotide polymorphisms (SNPs) loci in 2021 and 167 in 2022. Key candidate regulators included genes involved in lipid biosynthesis (*ATS2*, *KAS*, *LPP*), amino acid transport (*CAT4*) for protein storage, *zero tannins locus-1* (*zt-1*), and regulators of the phenylpropanoid pathway, such as a *shikimate kinase* gene and transcription factors *bHLH137-like* and *MYB*. These results pave the way for validation studies and biotechnological applications to improve the quality of faba bean-based foods.

## 1. Introduction

The plant-based food sector is rapidly growing due to environmental, ethical, and health concerns associated with the high consumption of animal-based products. Raising animals generally results in greater environmental impacts than cultivating plants, including higher greenhouse gas emissions, increased water and land use, and nitrogen pollution [1]. Ethical considerations and the perception of plant-based diets as healthier are also driving this shift [2,3]. Consequently, the European retail sales of plant-based foods grew by 21% between 2020 and 2022, reaching €5.8 billion [4].

The faba bean (*Vicia faba* L.) is gaining attention in the plant-based food industry for its high protein content and functional properties [5] as well as for its ability to fix nitrogen through symbiosis with *Rhizobium* bacteria, making it an environmentally friendly crop. Pulses like the faba bean are frequently used in products aiming to mimic animal-based foods such as milk, cheese, meat, fish, and eggs. The faba bean is increasingly incorporated into bread, pasta, snacks, and infant formulas [6,7,8]. Moreover, studies have shown that the faba bean protein concentrate is suitable for meat-like products [9], and fibrous textured meat analogues have been successfully produced [10].

However, consumer acceptance of faba bean-based products is hindered by “off-flavours”, defined as an unpleasant taste and/or aroma [11]. These off-flavours are problematic in plant-based food where consumers expect flavours similar to meat, cheese, milk, etc. Off-flavours are caused by volatile (aroma) or non-volatile (taste) compounds [12]. They are described as dried pea-like, beany, bitter, and with an unpleasant fruity note in faba bean [13,14]. Volatile compounds from lipid oxidation contribute significantly to off-flavours, especially those derived from polyunsaturated fatty acids (PUFAs) such as linoleic acid (C18:2) and linolenic acid (C18:3) [15]. PUFAs undergo enzymatic oxidation (via lipoxygenase) or autoxidation, producing volatiles with strong off-flavour properties [12]. Additionally, non-volatile PUFA-derived compounds (e.g., trihydroxy fatty acids, etc.) are bitter even at low concentrations, and PUFAs themselves are also bitter [16]. Other bitter and/or astringent compounds in legumes include polyphenols (phenolic acids, flavonoids, and tannins), saponins, amino acids, and peptides [17,18,19]. Tannins, in particular, contribute to bitterness or astringency in faba beans based on their degree of polymerization [20,21]. Although saponins are bitter and/or astringent in pea, their lower concentration in faba beans makes them unlikely major contributors [22]. Recently, the alkaloids vicine and convicine have also been proposed as contributors to bitterness and mouth dryness [22]. Breeding offers an alternative for reducing off-flavours to costly post-harvest treatments (e.g., thermal, chemical, or enzymatic), which are expensive and energy-intensive [12]. Contrarily, targeting the off-flavour precursors or enzymes involved in flavour development through breeding can address the issue upstream. Lippolis et al. [23] reviewed the challenges of breeding for reduced off-flavours in faba bean and proposed marker compounds, including volatile (e.g., hexanal) and non-volatile (e.g., 1-linoleoyl glycerol) lipid-oxidation products, phenolic acids (e.g., p-coumaric acid, ferulic acid), flavonoids (e.g., quercetin), and tannins (e.g., epicatechin, procyanidin B2).

Genetic research into off-flavour traits and protein content in faba bean has been limited. Previous studies have examined volatile off-flavours [24], phenolic acids and flavonoids [25], fatty acids, total phenols, total saponins, and total tannins [26,27]. However, these studies characterized only a limited number of samples (e.g., fewer than 50), and they lacked estimates of important genetic information such as heritability or genetic correlations across traits and/or environments.

Efforts to map quantitative trait loci (QTLs) controlling off-flavour and protein content in faba bean are also limited. Zhao et al. [28] mapped QTLs for lipid and protein content, but these did not co-localize with known biosynthesis genes. Similarly, Ohm et al. [29] attempted to map QTLs for protein content using a genome-wide association study (GWAS) but identified no candidate markers. To our knowledge, no attempts have been made to map phenolic acids and flavonoids in faba bean. In contrast, tannin, vicine, and convicine contents have been extensively studied, with QTLs and molecular markers available [30,31,32,33,34,35]. These traits have historically attracted research and breeding investment due to their role as anti-nutritional factors.

Further research is needed to explore the genetic diversity of off-flavours and protein content in faba bean and to identify molecular markers and candidate genes. No GWAS analyses have been reported for oil content, fatty acid composition, lipid-derived products, flavonoids, phenolic acids, and tannins. To address this gap, we dissected the genetic architecture of protein content and various off-flavour-related compounds using a GWAS. We assembled a diverse panel of 245 accessions, genotyped them using a Single Primer Enrichment Technology (SPET) assay, and phenotyped them in 2021 and 2022 in the Netherlands for protein, oil, fatty acids, lipid-derived compounds, phenolic acids, flavonoids, and tannins.

## 2. Results

### 2.1. A Large SNP Panel for GWAS

**Figure 1:**
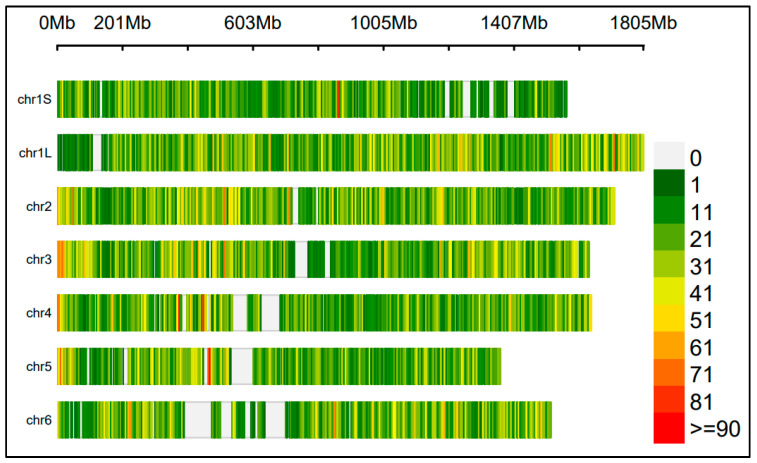
SNP coverage along the six chromosomes of faba bean (*Vicia faba* L.). Chromosome 6 had the largest gap among adjacent SNPs, followed by chromosome 5 and 4. The specific chromosome length represents the regions covered by the markers.

The CGN population was genotyped using the 90K-SPET assay, resulting in a high-quality SNP dataset with a median depth of coverage of 240x. The high coverage enabled the reliable estimation of allelic frequencies in pooled samples. After filtering, 48,445 SNPs were retained for the GWAS. The SNPs were well distributed across the genome, with an average marker density of ~4 SNPs per one million base pairs (1 Mbp). Chromosome 6 had the largest gap between adjacent SNPs, followed by chromosomes 5 and 4 (Figure 1). Detailed information on SNP counts per chromosome, average densities, and maximum gap distances is provided in Supplementary Table S1.

### 2.2. Reliable NIRS Prediction Models

Modified Partial Least Squares (MPLS) regression models were developed using NIRS and chemical data from the training sets. The aim was to predict the remaining samples based on NIRS data only using the developed models. The NIRS models were evaluated through 10-fold cross-validation. Good predictions were obtained for protein, oil, C18:1, C18:2, C18:3, catechin, epicatechin, procyanidin B1, and procyanidin B2, as indicated by the high Pearson correlation values (r_cv_ > 0.7) between the predicted and actual chemical values (Table 1). In contrast, predictions for phenolic acids, flavonoids, and lipid-derived products did not show robust correlations. Consequently, only the lab-generated chemical data on the training set were available for these compounds.

### 2.3. Large Phenotypic Variability

The data analysis of the field trials involved fitting single-trait and single-environment linear mixed models to estimate adjusted means. Overall, large phenotypic variability was observed in the CGN population in both years. Most traits had a coefficient of variation (CV%) greater than 10%, with particularly high values for tannins, phenolic acids, flavonoids, and lipid-derived products (Table 2). The only exception to the large CVs was C18:2, with a coefficient of variation (CV%) of ~2.7%.

Notably, some accessions showed (phenotypically) simultaneous low levels of complex tannins (procyanidin B1 and B2) and flavonoids (myricetin and quercetin) in both years, including CGN12310 (Canadian landrace), CGN13511 (Pakistani landrace), CGN15623 (Dutch variety), CGN15630 (Finnish variety), and CGN18933 (Israeli variety). Moreover, the desirable combination of high protein content and high C18:1 was found in both years in CGN07871 (Afghani landrace), CGN10333 (German variety), CGN10334 (German variety), CGN10335 (German variety), CGN12307 (Canadian landrace), CGN13457 (French variety), CGN15587 (Czechoslovakian variety), CGN15630 (Finnish variety), and CGN18893 (Belgian variety). These accessions are good candidates for further screening to breed for reduced bitterness, astringency, and beany flavour.

### 2.4. Trait-Specific Genotype-by-Year Interaction (GxY)

Multi-environment trial (MET) linear mixed models were fitted using a one-step approach, combining data from 2021 and 2022. The effect of cultivation year on phenotypic responses was evaluated using Wald test statistics. The extent of genotype-by-year interaction (GxY) was assessed using type-B additive genetic correlations.

The year of cultivation had a highly significant effect on all traits, with *p*-values ≤ 1.426 × 10^−¹⁰^, except for C18:2, which had a higher *p*-value of 0.014 but was still statistically significant.

The extent of GxY was trait-specific (Table 3). Overall, oil and fatty acid content (C18:0, C18:1, C18:2) were quite stable across the years, with ρ_type-B_ > 0.73. Tannins were also rather stable, with ρ_type-B_ ranging from 0.55 to 0.81; quercetin showed the highest stability (ρ_type-B_ > 0.82) among phenolic acids and flavonoids. Contrarily, protein, caffeic and p-coumaric acid, and myricetin were more affected by GxY, as indicated by ρ_type-B_ < 0.5.

### 2.5. High Heritability (h^2^) for Oil, Fatty Acids, Protein, and Tannins

The narrow-sense heritability (*h*^2^) estimated for each year varied among traits (Table 3). Overall, the *h*^2^ values for oil, fatty acids, protein, and tannins indicated strong genetic control on these traits in both 2021 and 2022 (0.45 < *h*^2^ < 0.84). In contrast, phenolic acids (p-coumaric, caffeic acid), flavonoids (myricetin, quercetin), and lipid-derived products (hexanal, 1-linoleoyl glycerol, 2-hydroxyoleic acid) exhibited moderate to low genetic control (0.19 < *h*^2^ < 0.49). Ferulic acid showed low heritability in 2021 and was not included in the GWAS analysis.

### 2.6. Specific Type-A Additive Genetic Correlations (ρ_type-A_) Between Traits

**Figure 2:**
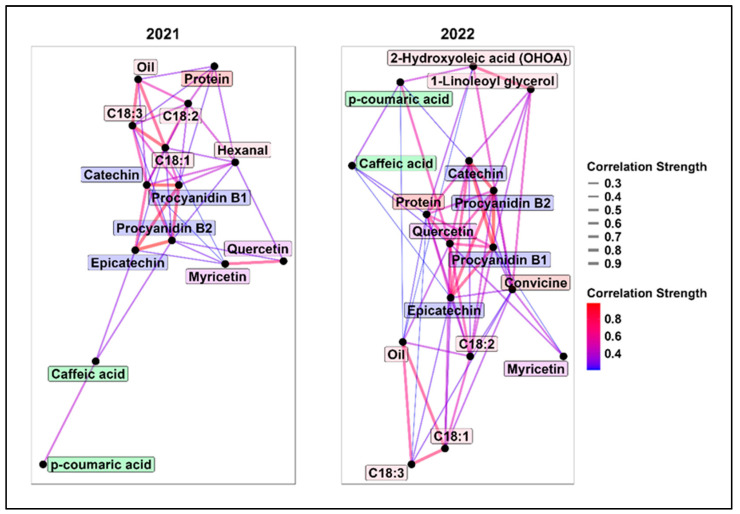
Correlation network based on type-A additive genetic correlations (ρ_type-A_) in 2021 (**left**) and 2022 (**right**). Only absolute ρ_type-A_ > 0.2 are displayed. Each node (black circle) represents a different trait, and each edge (line) represents the absolute genetic correlation among traits. As the legend indicates, thick red lines represent strong correlations, while thin blue lines represent weaker correlations. The labels’ background colour groups molecules according to their chemical classes.

Type-A additive genetic correlations were calculated using bivariate LMMs for each pair of traits in 2021 and 2022. Figure 2 shows a network of these correlations, including only those with absolute values greater than 0.2. Full correlation matrices and approximated standard errors are provided in Supplementary Tables S2 and S3.

Overall, tannin molecules showed high positive genetic correlations in both years, ranging from 0.65 to 0.98. Tannins were negatively correlated with protein, with stronger correlations observed in 2022 (−0.46 < ρ_type-A_ < −0.56).

Oil content was strongly positively correlated with C18:1 (ρ_type-A_ > 0.7) and negatively with C18:3 (ρ_type-A_ < −0.66). As expected, C18:1 and C18:3 were strongly negatively correlated (ρ_type-A_ < −0.8). The trade-off between oil and protein was minimal (ρ_type-A_ = −0.2).

In 2021, positive correlations between C18:1 and catechin (ρ_type-A_ = 0.54) and procyanidin B1 (ρ_type-A_ = 0.45) were less favourable for breeding, as high C18:1 and low tannins are preferred to reduce off-flavour. However, correlations were weaker in 2022.

Hexanal showed a moderate correlation (ρ_type-A_ = 0.38) with its precursor C18:2, while the other lipid oxidation products showed weak or no correlations with oil and fatty acids. 1-linoleoyl glycerol and 2-hydroxyoleic acid (OHOA) were strongly correlated (ρ_type-A_ > 0.7) and moderately correlated with convicine (ρ_type-A_ = 0.45).

In 2022, quercetin emerged as a key molecule, showing moderate to high genetic correlation with C18:2, protein, tannins, and p-coumaric acid.

### 2.7. Identification of SNPs via GWAS

The GWAS analysis using 48,445 SNPs was performed separately for 2021 and 2022. However, data were available for only one year for hexanal, 1-linoleoyl glycerol, and 2-hydroxyoleic acid (OHOA).

**Figure 3:**
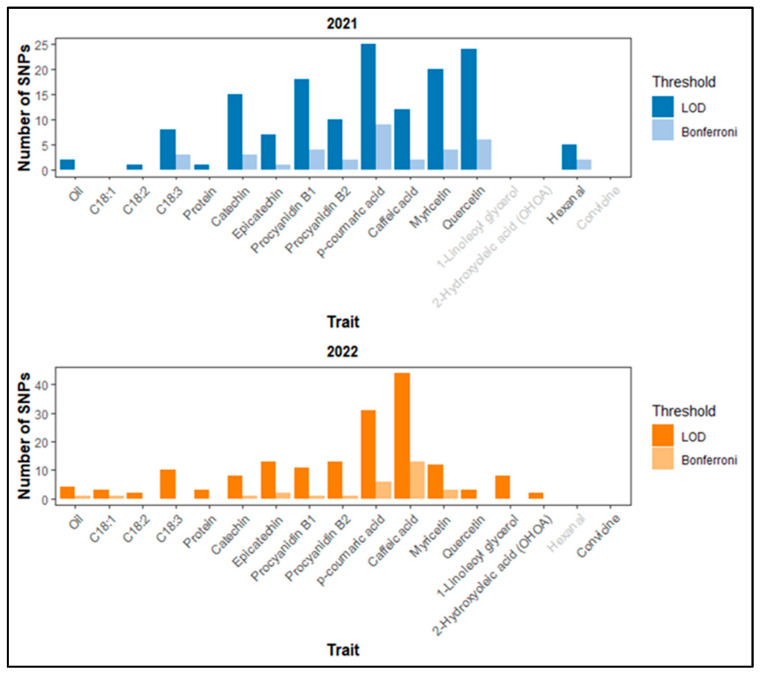
Number of SNPs detected by GWAS per trait in 2021 (**top**) and 2022 (**bottom**) based on LOD ≥ 4.5 and the Bonferroni correction threshold. Grey labels on the x-axis indicate no data available.

Based on the LOD threshold of 4.5, there were 148 SNP-trait associations in 2021 and 167 in 2022. Of these, 36 in 2021 and 29 in 2022 met the more stringent Bonferroni correction threshold (*p*-value 1.03 × 10^−6^) (Figure 3). No significant SNPs were detected for C18:1 in 2021 and for convicine in 2022. Notably, several SNPs individually explained more than 20% of the phenotypic variation (Supplementary Tables S4 and S5). Table 4 presents a subset of significant SNPs. We prioritized SNPs that are consistent across years, shared among multiple traits, or associated with candidate genes potentially involved in relevant biological pathways based on their annotations.

**Figure 4:**
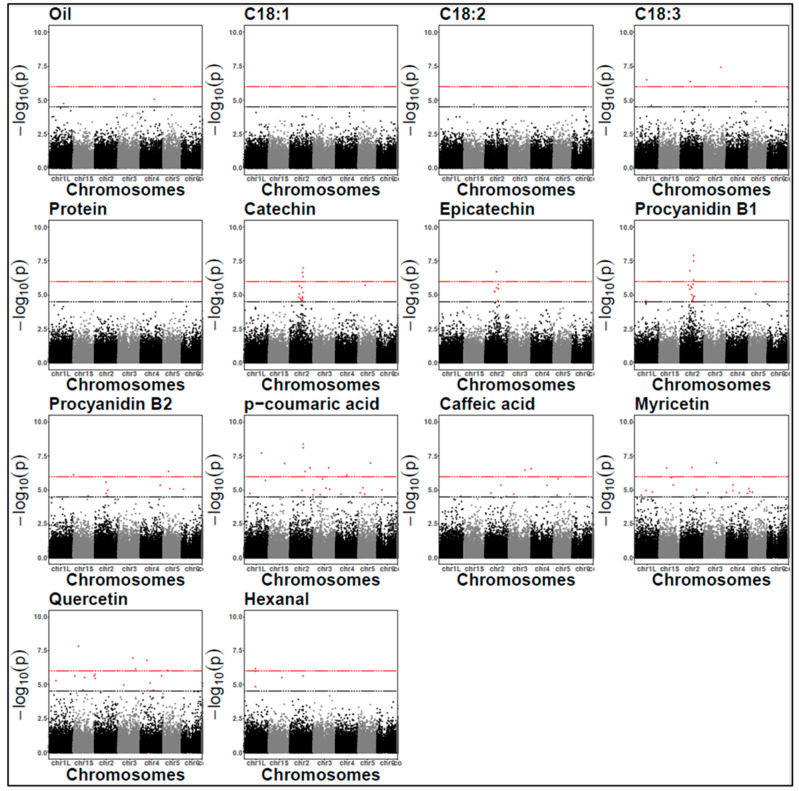
Manhattan plots for protein content and off-flavours (2021). The black dashed line represents the LOD threshold of 4.5, and the red dashed line indicates the Bonferroni threshold. Each dots represent a SNP. SNP locations on chromosomes are indicated on the x-axis.

**Figure 5:**
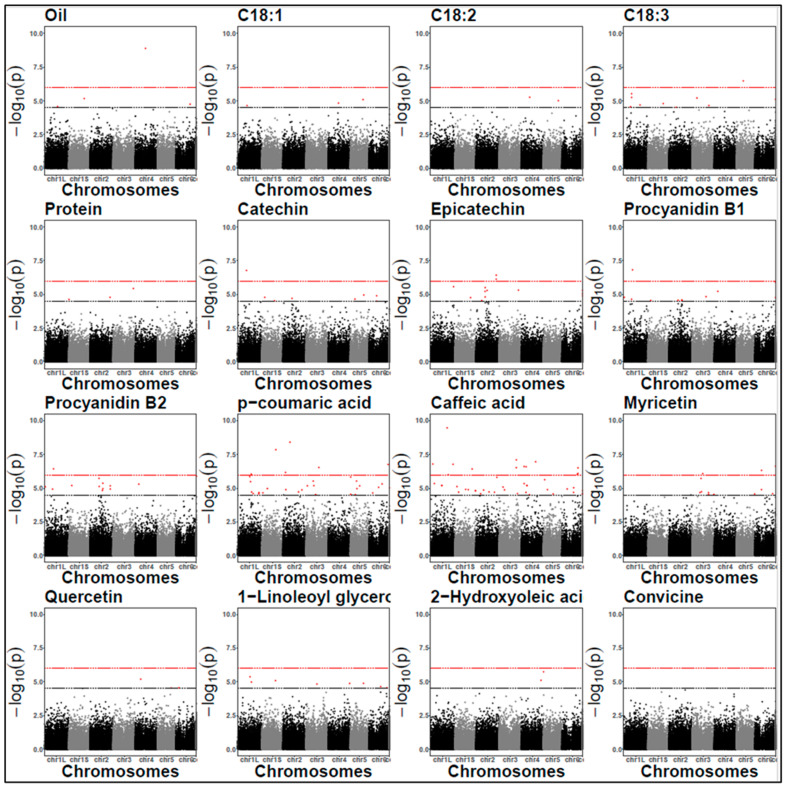
Manhattan plots for protein content and off-flavours (2022). The black dashed line represents the LOD threshold of 4.5, and the red dashed line indicates the Bonferroni threshold. Each dots represent a SNP. SNP locations on chromosomes are indicated on the x-axis.

The genomic inflation factors for each GWAS model were all very close to the expected values of one (Supplementary Table S6). The Manhattan plots for the association analysis are presented in Figure 4 and Figure 5, while the QQ plots are in Supplementary Figures S2 and S3.

Approximately 80% of the significant SNPs were intragenic in both years (Supplementary Tables S4 and S5), potentially pinpointing candidate genes through direct association. These intragenic SNPs were analysed for their predicted effects on the encoded proteins. In 2021, 34 SNPs resulted in amino acid substitutions (missense variants) compared to the 50 missense variants identified in 2022.

### 2.8. Candidate Genes

Several candidate genes involved in diverse biological functions were identified for all traits (Supplementary Tables S4 and S5), expect for convicine as no markers were significantly associated with this trait.

Interestingly, a *cationic amino acid transporter 4* (*CAT4*) gene was a candidate gene for protein content (Table 4). Genes involved in lipid metabolism pathways were candidates for oil and fatty acids. These included *lipid phosphate phosphatase* (*LPP/PAP*), *1-acyl-sn-glycerol-3-phosphate acyltransferase* (*ATS2/LPAAT*), *3-oxoacyl-[acyl carrier protein] synthase* (*KAS*), and a *lipase* gene (Table 4).

**Figure 6:**
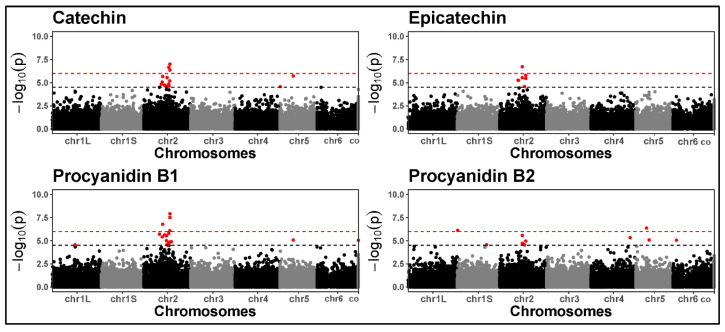
Manhattan plots for tannins based on the GWAS performed in 2021. The black dashed line represents the LOD threshold set at 4.5, while the red dashed lines represent the Bonferroni-corrected threshold. Significant signals on chromosome 2 spanned positions 671,378,841 bp to 953,872,991 bp. The x-axis labels “chr1L” and “chr1S” indicate the official division of the very large chromosome 1, as defined by the faba bean genome consortium.

Tannins were associated with multiple signals on chromosome 2 (Figure 6). We performed a BLAST analysis of the *Medicago truncatula* transcription factor WD-40 (*TTG1*) gene against the faba bean reference genome, which revealed a strong match within the significant region. This region harbours the *zero tannin-1* (*zt-1*) locus. Detecting the expected *zt-1* validated our GWAS pipeline.

The transcription factors bHLH and MYB were proposed as candidate regulators of the phenylpropanoid pathway involved in p-coumaric and caffeic acid biosynthesis. Additionally, two SNPs associated with caffeic acid were located within a *shikimate kinase* (*SK*) gene, potentially influencing phenolic compound production via the shikimate pathway (Table 4).

## 3. Discussion

Breeding for improved faba bean quality is primarily driven by the food industry’s demand to increase protein content and reduce off-flavours in plant-based products. This study aimed to investigate the natural variation present in the faba bean germplasm for these traits and dissect their genetic architecture via a GWAS.

Phenotyping chemical traits is typically costly, labour-intensive, and time-consuming. We successfully used NIRS to predict protein, oil, fatty acids, and tannins, achieving high cross-validation correlations (r_cv_ > 0.72) among the predicted and actual lab values. However, as cross-validation results may overestimate predictive performance, further validation on external samples or under different condition is necessary. Notably, this is the first successful NIRS-based prediction of C18:2, C18:3, and individual tannins in this crop. Further research should focus on predicting phenolic acids, flavonoids, and lipid-derived products. Poor predictions for these compounds in our study are likely due to their low concentrations below the NIRS detection threshold [36].

The CGN population exhibited substantial phenotypic diversity, as indicated by the high coefficients of variation (CV%). This diversity was expected due to the inclusion of three botanical groups (*Vicia faba minor*, *major*, and *equina*) from 43 countries and the low genetic relatedness between CGN accessions [37]. We identified accessions with C18:1 content up to 27% and protein content up to 35% (phenotypic values). These values are among the highest reported in the literature [29,38,39]. Thus, these accessions are promising breeding candidates, as high C18:1 and protein are key targets. Some genotypes also showed low levels of phenolic acids, tannins, flavonoids, and lipid-derived products. Overall, tannins were higher than flavonoids, which exceeded phenolic acids. This pattern was previously observed [25,40,41]. The lower abundance of phenolic acids is likely due to their rapid metabolism. This study is the first to report the presence of 1-linoleoyl glycerol and 2-hydroxyoleic acid in faba bean. These are compounds that were previously linked to bitterness in peas [42].

The heritability (*h*^2^) values suggest that genetic gain can be achieved faster for traits such as oil, fatty acids, and tannins, with *h*^2^ > 0.6 in both years. Protein content had high *h*^2^ in 2022 (0.7) but moderate *h*^2^ (0.45) in 2022, likely due to the better field conditions. For phenolic acids, flavonoids, lipid-derived products, and convicine, *h*^2^ values ranged from moderate to low (0.07 < *h*^2^ < 0.49). For these compounds, separating genetic signals from residual noise is challenging due to the lower replication, so the *h*^2^ estimates mainly reflect the few genotypes with field replicates.

The year of cultivation significantly affected all chemical traits. In 2022, higher protein content was likely due to better field conditions and nitrogen availability, while higher levels of phenolic acids, flavonoids, and tannins in 2021 were likely driven by biotic and abiotic stress pressure [43,44]. Previous research has shown that cultivation location or year affects volatile compounds [45], oil [46], total phenolics and tannins [47], and protein [46], all of which also exhibited genotype-by-environment (GxE) interactions.

Different genotypes respond differently to environmental changes—a behaviour known as GxE. High additive type-B genetic correlations (ρ_type-B_ > 0.7) for oil, fatty acids, and tannins suggest low genotype-by-year (GxY) interactions, supporting the development of stable varieties in the Netherlands. In contrast, a ρ_type-B_ of 0.5 for protein indicates challenges in breeding high-protein varieties suited for diverse environments. Among phenolic acids and flavonoids, quercetin was the only relatively stable trait (ρ_type-B_ = 0.82). Although limited to two years, this study advances the understanding of GxY interactions for key chemical traits in faba bean.

Improving faba bean for plant-based food requires selecting multiple traits. High additive type-A genetic correlations (0.65 < ρ_type-A_ < 0.98) between catechin, epicatechin, procyanidin B1, and procyanidin B2 suggest shared genetic control, aligning with their common biosynthetic pathway [48]. These high correlations simplify selection as one molecule can serve as a proxy for others. Low tannin levels do not significantly hinder breeding for high protein, given their weak type-A correlations (−0.3 < ρ_type-A_ < −0.1). This aligns with the findings of Walter et al. [49], who suggested a natural trade-off between nitrogen- and carbon-based compounds. Additionally, no strong negative genetic correlations were found between tannins, phenolic acids, and flavonoids, allowing tannin reduction without increasing other compounds given the lack of opposing genetic control.

Breeding for high C18:1 stabilizes oil and reduces off-flavours, as we discussed in our previous research [23]. The moderate to strong ρ_type-A_ between C18:1 and C18:2 and C18:3 are positive and expected findings [50]. The strong positive correlation between oil and C18:1 (ρ_type-A_ > 0.7) suggests that selecting for C18:1 via oil content offers a cost-effective alternative to gas chromatography (GC).

Positively, oil showed a negligible trade-off with protein (ρ_type-A_ = −0.2), enabling genetic gains in both traits. This weak correlation is expected in faba bean, unlike high-oil accumulator legumes like soybean, which often exhibits a strong negative genetic correlation between oil and protein [51]. However, the moderate positive genetic correlation between C18:1 and certain tannins should be considered when breeding for high C18:1.

### Novel QTLs and Candidate Genes

The GWAS-detected SNPs offer initial insights into key molecular markers and candidate genes for validation in replicated studies or marker-assisted selection (MAS). Targeted genotyping (90K-SPET) produced one of the largest SNP panels (48,445 SNP) available in faba bean. Approximately 80% of significant SNPs were located within genes, facilitating the discovery of candidate genes or causal variants. However, not all intra-genic SNPs directly impact gene function or represent the gene itself, and it is pivotal to investigate genes harboured in the LD blocks. About 30% of the intragenic SNPs were predicted to alter protein sequences (missense variants), warranting validation in functional studies to assess their effects on proteins and phenotypes. We identified intra-genic SNPs for validation as functional variants in future studies, with ~30% predicted to alter protein sequences (missense variants). We also identified interesting genes in the LD blocks.

Ohm et al. [29] attempted to dissect the genetic architecture of protein content via a GWAS but detected no QTLs. Herein, we report four SNPs associated with protein content, albeit with minor effects, supporting the notion that many small-effect QTLs control protein content. One SNP identified in 2021 (SNP chr5_632641479) was located near a *cationic amino acid transporter 4* (*CAT4*) gene. Amino acid transporters contribute to nitrogen partitioning among source and sink tissues in plants [52]. Although *CAT4* has not been directly linked to protein storage, research on another amino acid transporter subfamily in faba bean suggests that *Acid Permease* (*AAP1*) supplies amino acids for synthesizing storage proteins [53]. *CAT4* may function similarly, providing amino acids used for protein storage. This is supported by studies showing that *CAT4* mutants increased histidine levels in *Arabidopsis* seeds [54].

Consistent with the high additive genetic correlations (type-A and type-B), the GWAS identified stable genetic signals across the years for C18:3 (SNP chr1L_1230530421, SNP chr3_1234617141, SNP chr5_505151579, and SNP contig_7845_77914). Shared genetic control was identified between oil content and C18:3 (SNP chr1S_1143102257) and between oil content and C18:1 (SNP chr4_755718730). The detected SNPs were in linkage disequilibrium with lipid biosynthesis genes, including *lipid phosphate phosphatase* (*LPP/PAP*), *1-acyl-sn-glycerol-3-phosphate acyltransferase* (*ATS2/LPAAT*), *3-oxoacyl-[acyl carrier protein] synthase* (*KAS*), and a lipase gene. Interestingly, *ATS2/LPAAT* and *LPP/PAP* act sequentially in lipid biosynthesis. First, *ATS2/LPAAT* converts lysophosphatidic acid (LPA) to phosphatidic acid (PA). Then, *LPP/PAP* dephosphorylates PA to diacylglycerol (DAG). DAG is a key intermediate for triglyceride (TAG) and phospholipid synthesis [55]. Alterations in *ATS2* and/or *LPP* activity may affect fatty acid amounts and ratios by altering the PA/DAG balance. In *Arabidopsis*, *LPP* overexpression or suppression alters fatty acid composition, suggesting that *LPPs* may promote TAGs enriched with polyunsaturated fatty acids [56]. Additionally, the candidate gene *KAS* is essential for fatty acid chain elongation by catalysing β-ketoacyl-ACP synthesis and providing acyl chains for lipid synthesis [57]. Our findings highlight the complex regulation required to maintain proper lipid composition in eukaryotic cells. However, these candidate genes require further validation in faba bean to confirm their roles, expression patterns, and locations.

Tannins were associated with stable signals across the years, consistent with the high type-B genetic correlations. Specifically, SNP chr2_671378841 (catechin, epicatechin, procyanidin B1), SNP chr2_826275103 (epicatechin), and SNP chr2_953872991 (procyanidin B1, procyanidin B2) indicate a strong genetic control of multiple tannins on chromosome 2. The region defined by these significant SNPs (spanning positions 671,378,841 bp to 953,872,991 bp) harbours the *zt-1* (*zero tannin-1*) gene, an orthologue of *Medicago truncatula’s TTG-1* (*transparent testa glabra-1*). *TTG-1* is already known to regulate tannin biosynthesis in faba bean [30]. Despite detecting a clear and expected signal, no significant SNPs directly flank *zt-1* within the ~268 kb linkage disequilibrium (LD) window, calculated as a genome-wide average [37]. The several significant SNPs detected within this extensive region suggest an extended LD in this area, possibly due to historical selection pressure for low tannin content due to their anti-nutritional properties.

This study is the first to map the genetic control of phenolic acids and flavonoids in faba bean. However, a limitation was the relatively small sample size due to missing data from poor NIRS predictions. Although phenolics and flavonoids both belong to the phenylpropanoid pathway, no shared QTLs were identified except for SNP chr4_935741377, associated with both myricetin and quercetin in 2021. The lack of stable QTLs across the years hinders robust marker-assisted selection for these traits. Positively, SNPs chr6_1188716500 and SNP chr6_1188716505, associated with caffeic acid in 2022, were located within a *shikimate kinase* (*SK*) gene. This gene is crucial for producing phenylalanine—a precursor of these phenolic compounds. Yuan et al. [58] showed that silencing the *PhSK* gene alters flavonoid metabolism and reduces anthocyanin content in *Petunia*. We could hypothesize that SK protein alterations may reduce caffeic acid and other polyphenols in crops, but further validation is needed.

Our analysis confirmed that members of the bHLH and MYB transcription factors family play a role in phenylpropanoid pathways [59]. SNP chr2_964372547 and chr2_964372565, associated with p-coumaric acid in 2021, were located near a *bHLH137-like* gene. Transcriptome analyses in carrot and ornamental cabbage revealed that *bHLH137-like* expression correlates with anthocyanin content and purple pigmentation [60,61], suggesting it may regulate the phenylpropanoid pathway from which anthocyanin and p-coumaric acid originate. In 2022, SNP chr1S_1040083079 was located near a gene encoding a protein with domains typical of MYB transcription factors, emphasizing the complexity of transcriptional regulation in the phenylpropanoid pathway.

For the first time in faba bean, we report the candidate genes involved in the control of volatile lipid-derived products such as hexanal and non-volatile lipid-derived products such as 1-linoleoyl glycerol and 2-hydroxyoleic acid (OHOA). Since these genes are not directly linked to the expected oxidation pathways, their roles need to be verified further.

## 4. Material and Methods

**Figure 7:**
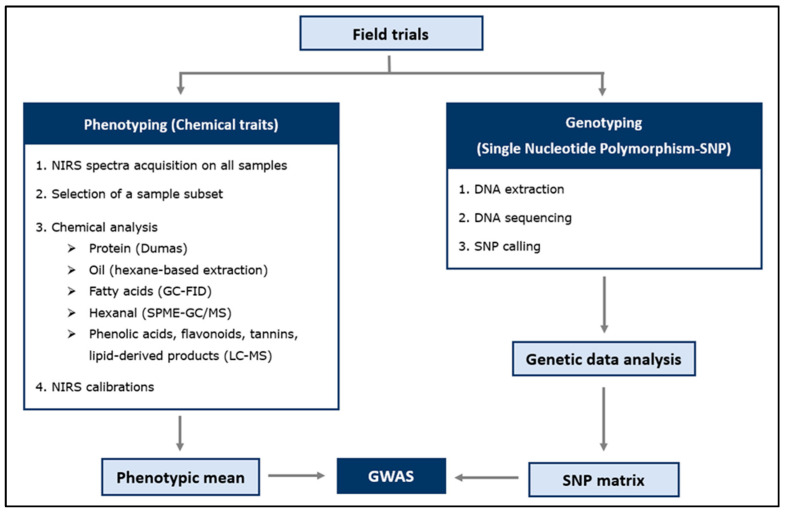
Faba bean plants were grown for two years in field trials in the Netherlands. Two datasets were generated: phenotypic (chemical) and genotypic (SNP) data. Phenotypic data: a subset of samples was chemically analysed (training set), while the remaining samples (field plots) were predicted using Near-Infrared Spectroscopy (NIRS). Both phenotypic and genotypic data were used to perform a genome-wide association study (GWAS).

A schematic workflow of the material and methods of this study is summarized in Figure 7.

### 4.1. Plant Material and Experimental Design

The plant material, experimental design, and field trial management for this study were previously detailed in Lippolis et al. [37]. Briefly, the CGN population comprised 245 faba bean accessions collected from the Centre for Genetic Resources (CGN) in the Netherlands (NL) and six commercial varieties from Limagrain and NPZ. The plants were evaluated in Winschoten (NL) for two consecutive years (2021 and 2022) using a resolvable row-column design (RRC). The design was generated with the FielDHub R-package [62], including two complete blocks, each with 11 rows and 25 columns. Data were recorded on the six central plants in a 1.8 m^2^ plot, which contained 20 plants each.

### 4.2. Phenotyping: NIRS (Near-Infrared Spectroscopy) and Chemical Analysis

The compounds analysed were protein, oil, fatty acids (oleic acid C18:1, linoleic C18:2, and linolenic acid C18:3), lipid-derived products (hexanal, 1-linoleoyl glycerol, and 2-hydroxyoleic acid (OHOA)), phenolic acids (p-coumaric, caffeic, and ferulic acid), flavonoids (myricetin and quercetin), tannins (catechin, epicatechin, procyanidin B1, and procyanidin B2). All the commercial standards used in the analysis were obtained from Sigma-Aldrich.

Hexanal was chosen as an effective marker for lipid oxidation reactions. 1-linoleoyl glycerol, 2-hydroxyoleic acid, phenolic acids, flavonoids, and tannins were selected due to their known bitter and astringent properties [12,23].

#### 4.2.1. NIRS Spectra Acquisition

NIRS data were obtained as described by Lippolis et al. [63]. Briefly, ground samples (particle size < 0.5 mm) were scanned separately after each of the two harvests using a DS2500 Analyzer (VIS-NIR) spectrometer (FOSS Analytical A/S, Hillerød, Denmark). The data represented absorbance spectra (log 1/reflectance) at a 2 nm resolution from 400 to 2500 nm. After spectra collection, the samples were stored at −18 °C until further chemical analysis.

#### 4.2.2. Sub-Sample Sample Selection (Training Set)

A subset of samples (training set), used to perform chemical analysis and develop the NIRS prediction models, was selected based on spectral diversity. This approach included data pre-processing, principal component analysis (PCA), outlier detection, and algorithm comparisons for training set selection, which followed the methodology detailed in Lippolis et al. [63].

In short, a two-step selection process was used to select samples for protein, oil, C18:1, C18:2, and C18:3. First, 125 samples were selected using K-means clustering from the 2021 trial. Subsequentially, 67 samples were selected from the 2022 trial using the Kennard–Stone algorithm. The selected samples were combined into a single training set that underwent chemical analysis. Only the first selection step (125 samples in 2021) was applied for phenolic acids, flavonoids, tannins, and lipid-derived products, resulting in 125 samples selected from the 2021 trial. These same samples were re-analysed in 2022.

#### 4.2.3. Chemical Analysis of Protein, Oil, and Fatty Acids

The chemical data for protein, oil, and C18:1 were obtained as described by Lippolis et al. [63]. Briefly, protein content was measured in duplicate using the Dumas method with a standard conversion factor of 6.25. Oil content was determined in triplicates using a hexane-based extraction. The C18:1 content was analysed as a Fatty Acid Methyl Ester (FAME) using gas chromatography (GC) with a Flame Ionization Detector (FID) (GC-FID, Agilent Technologies, Santa Clara, USA). The same protocol used in Lippolis et al. [63] was applied for C18:2 and C18:3, utilizing their respective FAME commercial standards. Samples were run in duplicates and quantified based on peak area ratios obtained from the GC-FID analysis.

#### 4.2.4. Gas Chromatography-Mass Spectrometry (GC-MS) Measurements of Volatile Compounds (VOCs)

Solid Phase Micro Extraction (Arrow)—Gas Chromatography/Mass Spectrometry (SPME (Arrow)-GC/MS) was used to extract and analyse the volatile compounds in the headspace of the ground faba beans. Each sample (0.1 g powder) was mixed with 1 g NaCl (Merck), 0.15 mL 1M sodium phosphate monobasic and sodium phosphate dibasic buffer (Merck), and 5 mL demineralized water (final pH 7.1) in a 20 mL ND20 headspace glass vial (BGB^®^). The internal standard solution containing deuterated hexanal (0.5 μg/L) was added for quality control. A blank sample was prepared similarly. All samples were kept at 4 °C for at least 2 h to enable equilibrium.

Samples were incubated at 40 °C for 12 min with agitation (1200 rpm). Then, volatile extraction from the headspace was performed using a Restek PAL SPME-Arrow fiber DVB/C-WR/PDMS (120 µm × 20 mm) for 10 min at 40 °C with agitation (1200 rpm) via a TriPlus RSH autosampler (Thermo Scientific, Waltham, MA, USA). Volatiles were transferred to a Trace1300 gas chromatograph (Thermo Scientific, USA) coupled to an ISQ7000 single quadrupole mass spectrometer (Thermo Scientific, USA) by thermal desorption in a GC cryogenic cold trap (CryoFocus-4, GL Sciences, Eindhoven, The Netherlands) in split-less mode under constant hydrogen flow (2.17 mL/min). A Rxi-5SIL MS column (30 m × 0.25 mm ID × 1.0 µm film thickness) was used (Restek, USA). The GC oven temperature was started at 40 °C for 5 min, ramped up to 30 °C/min to 250 °C, and held for 2 min. The column effluent was ionized by electron impact (70 eV) with a scan range of *m*/*z* 25–250, and the MS interface was set at 260 °C.

Raw data were processed and analysed using Chromeleon 7.3.1 (Thermo Scientific, USA) and MsXelerator™ software 4.9.6 (MS Metrix, Utrecht, The Netherlands). Volatile compound identification was performed by matching mass spectra with the NIST17Spectral library. Integrated peak areas were adjusted for changes in MS detection sensitivity using the internal standards. Hexanal was quantified in ppm.

#### 4.2.5. Liquid Chromatography-Mass Spectrometry (LC-MS) of Non VOCs

Acetonitrile, methanol, and formic acid (ULC/MS grade) were obtained from Biosolve (Valkenswaard, The Netherlands). Calibration lines were prepared in an extraction solvent—a mixture of methanol and water 1:1 (*v*/*v*) acidified with 1% formic acid—according to Supplementary Methods-Table S7 and were used for quantification.

Each 500 mg ground sample was extracted with 10 mL extraction solvent and subjected to ultrasonication for 1 h. Clear extracts were obtained by centrifugation at 13.300× rpm at 4 °C. Each extract was measured in triplicate, and a pooled QC sample was measured after every ten samples.

All Liquid Chromatography-High Resolution Mass Spectrometry (LC-HRMS) analyses were performed using an UltiMate 3000 RS (Thermo Fischer Scientific, Waltham, MA, USA) connected to a Q-Exactive Plus Quadrupole-Orbitrap (Thermo Fischer Scientific, Waltham, MA, USA). Analytes were separated on a BEH C18 column (2.1 mm × 100 mm, 1.7 µm; Waters, Etten-Leur, The Netherlands) with the pre-column eluted at 0.30 mL/min using a linear gradient of water (A) and acetonitrile (B) containing 0.1% formic acid. The gradient was as follows: 0.1% B (1 min), 40% B (11.5 min), 100% B (2.5 min), and held at 100% B (2.5 min). The column and autosampler temperatures were at 40 °C and 5 °C, and the injection volume was 5 μL. Using default ionization source settings, the MS was operated in full-scan MS mode (70k resolution) in both positive and negative ionization modes. MS scans were acquired from *m*/*z* 100–1500, and additional analyses for identification were performed in ddMS2 mode (top 10) at 17.5k resolution with 2 *m*/*z* isolation windows and normalized stepped collision energies of 15, 30, and 60. Quantification was performed using Xcalibur 3.1 (Thermo) using the retention times and *m/z* values of Supplementary Methods-Table S8. All the compounds were quantified in ppm.

#### 4.2.6. Predictive Models Based on NIRS

Modified Partial Least Square (MPLS) [64] was used to model the linear relationship between spectra and chemicals, as implemented in WinISI^TM^ 4 software. Raw spectra were pre-processed using a Standard Normal Variate (SNV) coupled with Detrend (DT), and the mathematical treatment “1,4,4,1” was applied. Here, the numbers represent the derivative order (1), the gap over which the derivative is calculated (4 data points), the number of data points for smoothing (4), and the smoothing (1).

The optimal number of latent variables (LVs) was determined via 10-fold cross-validation in WinISI^TM^ 4 software. The 10-fold cross-validation splits the data into ten parts—training on nine and testing on one—and is repeated 10 times with each part used as the test set once. Model performance was primarily evaluated using the Pearson correlation coefficient (r_cv_) between predicted and actual chemical values of cross-validated samples.

Multi-year NIRS models for protein, oil, C18:1, C18:2, and C18:3 used a combined 2021–2022 training set. Year-specific models were developed for the other compounds.

### 4.3. Genetic and Phenotypic Data Analysis

#### 4.3.1. DNA Extraction, Sequencing, and SNP Typing

DNA extraction, sequencing, and SNP typing are detailed in Lippolis et al. [37]. Briefly, young lyophilized leaves from ten plants per accession were pooled to capture the intra-accession genetic variation. DNA was extracted using a NucleoMag^®^ DNA kit (Bioké, Leiden, The Netherlands). Sequencing was performed by IGAtech on an Illumina NovaSeq 6000 system (2 × 150 bp) using 90k-SPET^®^ technology.

After processing raw sequencing reads and variant calling [37], allelic frequencies (s) at each variant locus were calculated with a custom Python script as: $s=\frac{A_{r}}{A_{r}+A_{a}}$, where *A_r_* and *A_a_* are the reads counts of the reference and alternative alleles, respectively. Variants with insufficient coverage (average coverage <100×) were excluded for the robust estimation of *s*. Allelic frequencies were used in the GWAS after filtering for a minor allele frequency (maf) and ≥0.04 and ≤10% missing values using a custom R script. Missing values (Supplementary Figure S1) were imputed using Random Forest Imputation in the missForest R package [65], following the same approach as Nazzicari et al. [66].

#### 4.3.2. Genomic Relationship Matrix (G) and Principal Component Analysis (PCA)

The G matrix was calculated using an adapted Van Raden method for pooled data in the AGHmatrix R package [67] and blended with an identity matrix to ensure invertibility as already described in Lippolis et al. [37]. Following a PCA, K-means clustering identified four genetic clusters using information from the four retained principal components [37]. These four clusters were used as fixed effects in the GWAS models.

#### 4.3.3. Field Trial Analysis: Adjusted Mean, Heritability, Type-B, and Type-A Additive Genetic Correlations

Phenotypic (chemical) data were analysed using linear mixed models (LMM) fitted with the ASReml 4.2-R package [68]. All models are described in Appendix A. LMMs were fitted to estimate the narrow sense heritability (*h*^2^), adjusted means for each genotype (Y_adj_), type-A additive genetic correlations (ρ_type-A_) between traits [69], and type-B additive genetic correlations (ρ_type-B_) between years [69]. The narrow sense heritability (*h*^2^) was calculated after fitting generic single-trait and single-environment models as follows: $h^{2}=1-\frac{\left[mean\left(PEVg\right)\right]}{\sigma_{g}^{2}}$ [70], where *mean (PEV_g_)* represents the mean prediction error variance (PEV) for genotypes, and *σ_g_*^2^ denotes the estimated additive genetic variance. The additive genetic effects were estimated by using the Genomic Relationship Matrix (G), described earlier. The type-A and type-B additive genetic correlations were estimated as multi-trait (bi-variate) and multi-year LMM parameters, respectively. In detail, ρ_type-A_ and ρ_type-B_ were obtained by specifying heterogenous variance-covariance structures. A value of ρ_type-B_ close to one suggests minimal genotype-by-year (GxY) interactions. The significance of the year as a covariate (fixed effects) was assessed by the incremental Wald statistics, as reported in Model (A2) and described in Appendix A. Standard errors (approximated) for ρ_type-B_ and ρ_type-A_ were estimated using the Delta method [71].

#### 4.3.4. Linkage Disequilibrium Decay

Linkage disequilibrium (LD) decayed in the CGN population at approximately ~268.79 Kbp, as previously estimated by Lippolis et al. [37]. LD decay here refers to the average physical distance at which $r^{2}$ is halved from its maximum value (LD_max1/2_), which was calculated across all chromosomes. However, to avoid limiting the chances of identifying interesting candidate genes, it was taken into account that $r^{2}$ = 0.2 extends up to ~1000 Kbp in this population.

#### 4.3.5. Genome-Wide Association Study (GWAS)

The GWAS was performed using a linear mixed model (LMM) in the statgenGWAS R package [72] as follows: **y** = ***Xβ*** + ***Zu*** + ***e***, where ***y*** is the vector of Y_adj_, ***X*** is the design matrix for fixed effects (intercept, SNPs, and the four genetic cluster membership), ***β*** is the vector of fixed effect coefficients, with *β_SNP_* being the SNP-effect, ***Z*** is the incidence matrix, ***u*** is the vector of random genetic effects (additive) captured by the **G** matrix, with *var(****u****) = σ_g_*^2^***G***, and ***e*** is the vector of residual errors, with *var(**e**) = σ_e_*^2^***I***. StatgenGWAS estimated *σ_g_*^2^ and *σ_e_*^2^ using the Efficient Mixed Model Association (EMMA) algorithm [73]. Model fit was assessed with genomic inflation factors. SNPs with a LOD > 4.5 were deemed significant, and the Bonferroni threshold was used as a conservative reference for significance.

#### 4.3.6. Candidate Gene Identification and Putative SNP Effects

The candidate genes near significant SNPs were identified based on the LD decay. Gene annotations were obtained from publicly available annotations of the *faba bean* CV. *Hedin* reference genome. SNPeff tool [74] was used to predict the effect of intra-genic SNPs on proteins, categorizing impacts as “high” (disruptive), “moderate” (potentially altering protein function), “low” (likely harmless), or “modifier” (affecting non-coding regions or genes).

## 5. Conclusions

This study is the first to use a GWAS in faba bean to dissect the genetic architecture of chemical compounds related to seed quality and off-flavours. We provided key breeding insights, including heritability estimates and genetic correlations (type-A and type-B), facilitating efficient multi-trait selection strategies. We identified 148 SNP loci for 14 traits in 2021 and 167 SNP loci for 15 traits in 2022. The detection of intragenic SNPs and short linkage disequilibrium decay (~268 Kbp) revealed promising candidate genes. Notably, we reported missense variants that may be causal polymorphisms affecting phenotypes. This research paves the way for validation studies and applications to improve seed quality and reduce off-flavours in faba bean.

## Figures and Tables

**Table 1:** Person correlation between predicted and actual chemical values (r_cv_) of the 10-fold cross-validation (CV).

Trait ^a^	Cross-Validation	
	Tuning Parameter LVs ^b^	r_cv_ ^c^
Protein 2021–2022	12	0.99
Oil 2021–2022	10	0.97
C18:1 2021–2022	11	0.87
C18:2 2021–2022	11	0.82
C18:3 2021–2022	11	0.94
Catechin 2021	6	0.78
Catechin 2022	5	0.80
Epicatechin 2021	5	0.76
Epicatechin 2022	5	0.81
Procyanidin B1 2021	6	0.86
Procyanidin B1 2022	5	0.84
Procyanidin B2 2021	5	0.72
Procyanidin B2 2022	5	0.82

^a^ For protein, oil, fatty acids, a multi-year model was developed. ^b^ LVs indicates the number of latent variables (known as “terms” in WinISITM 4 software). The optimal number of variables was the one minimizing CV errors in WinISITM. ^c^ r_cv_ represents the average value across the 10 rounds of CV.

**Table 2:** Summary statistics for the adjusted phenotypic data of 245 faba bean (*Vicia faba* L.) accessions. The table details the minimum (min), maximum (max), mean, and coefficient of variation in percentage (CV%) in 2021 and 2022.

		2021				2022			
Chemical Class	Trait ^a^	Min	Mean	Max	CV%	Min	Mean	Max	CV%
Oil	Oil	1.37	1.69	2.14	7.93	1.23	1.49	1.84	7.07
Protein	Protein	18.70	25.14	30.66	7.91	22.02	29.29	35.15	7.04
Fatty acid	C18:1	15.74	21.17	27.11	11.19	14.81	20.05	25.12	9.23
	C18:2	48.68	52.73	56.60	2.77	48.51	52.97	58.83	2.88
	C18:3	2.50	4.79	7.37	15.15	2.57	5.20	7.40	12.60
Tannin	Catechin	1.04	41.93	80.17	38.28	0.28	7.22	15.51	42.20
	Epicatechin	0.15	37.04	70.01	39.69	1.23	11.76	21.63	37.58
	Procyanidin B1	1.81	77.85	121.08	33.10	0.51	16.14	31.10	37.33
	Procyanidin B2	0.22	90.60	224.26	32.37	1.42	32.20	60.16	37.16
Phenolic acid	p-coumaric acid	0.05	7.16	27.45	71.03	0.01	0.06	0.17	53.00
	Caffeic acid	0.04	1.46	5.58	72.98	0.02	0.56	1.97	61.30
	Ferulic acid	0.12	5.01	8.64	34.36	-	-	-	-
Flavonoid	Myricetin	0.01	14.98	62.09	96.12	0.06	4.92	18.84	86.01
	Quercetin	0.13	6.19	32.00	90.06	0.30	2.63	6.62	55.37
Lipid oxidation product	Hexanal	16.67	984.70	2797.92	62.36	-	-	-	-
	1-Linoleoyl glycerol	-	-	-	-	0.01	0.19	0.62	65.84
	2-Hydroxyoleic acid (OHOA)	-	-	-		2.37	7.18	10.87	27.08
Alkaloid	Convicine	-	-	-	-	2.31	16.67	28.42	27.90

^a^ Oil, protein, and fatty acids are expressed as a percentage of dry weight (%), while the other compounds are expressed in ppm.

**Table 3:** Type-B additive genetic correlations (ρ_type-B_) and year heritability (*h*^2^ _PEV_).

		2021	2022
Trait	ρ_type-B_ ^a^	*h*^2^ _PEV_ ^b^	*h*^2^ _PEV_
Oil	0.74 (0.06)	0.65	0.77
C18:1	0.81 (0.05)	0.74	0.78
C18:2	0.73 (0.08)	0.60	0.70
C18:3	0.87 (0.04)	0.74	0.82
Protein	0.5 (0.12)	0.45	0.70
Catechin	0.64 (0.07)	0.68	0.80
Epicatechin	0.81 (0.04)	0.72	0.87
Procyanidin B1	0.74 (0.05)	0.75	0.84
Procyanidin B2	0.55 (0.06)	0.75	0.84
p-coumaric acid	0.34 (0.13)	0.43	0.41
Caffeic acid	0.53 (0.10)	0.48	0.43
Ferulic acid	-	0.07	-
Myricetin	0.44 (0.10)	0.43	0.48
Quercetin	0.82 (0.14)	0.49	0.19
Hexanal	-	0.33	-
1-Linoleoyl glycerol	-	-	0.26
2-Hydroxyoleic acid (OHOA)	-	-	0.39
Convicine	-	-	0.42

^a^ The values in parentheses correspond to the approximated standard error. ^b^ *h*^2^ _PEV_ represents the narrow sense heritability, estimated based on the predictor error variance (PEV).

**Table 4:** Subset of significant SNPs detected by GWAS and their associated candidate genes. The table includes information on the chemical compound (trait) belonging to a specific chemical class (Compound Class), the year in which the SNP was detected (Year), the SNP identification code (SNP ID), the major allele frequency (allFreq), the raw *p*-values (*p*Value), the indication of whether the Bonferroni threshold was passed (Bonf), the effect of the SNP on the trait value (Effect), percentage of phenotypic variance explained by the SNP (Variance%), the SNP’s location relative to genes and the type of variant it represents (SNP location), the effect of the SNP on the encoded proteins (Protein impact), and the candidate gene annotation (Annotation).

Compound Class	Trait	Year	SNP ID ^a^	allFreq	*p* Value	Bonf	Effect	Variance (%)	SNP Location	Protein Impact	Candidate Gene Annotation ^b^
Lipid	Oil C18:3	2022	chr1S 1143102257	0.93	6.77 × 10^−6^ 1.59 × 10^−5^	no	−0.09 0.44	7.11 4.8	Intergenic region	Modifier	Cytochrome p450
	Oil C18:1	2022	chr4 755718730	0.91	1.27 × 10^−9^ 1.46 × 10^−5^	yes	−0.11 −1.18	15.11 6	Synonymous variant	Low	Cytochrome p450
	C18:1	2022	chr1L 713263969	0.94	2.25 × 10^−5^	no	−1.31	5.38	Synonymous variant	Low	Tryptophan aminotransferase protein Lipase in LD (~227.7 kbp)
	C18:3	2021/ 2022	chr1L 1230530421	0.94	2.46 × 10^−5^/ 2.02 × 10^−5^	no	−0.57/−0.43	5.1/4.1	Missense variant	Moderate	GDT protein 1 chloroplastic
	C18:3	2021/ 2022	chr3 1234617141	0.96	3.70 × 10^−8^/ 2.2 × 10^−5^	yes	−0.76/−0.49	8.3/4	Upstream gene variant	Modifier	Ras protein rab
	C18:3	2021/ 2022	chr5 505151579	0.94	1.27 × 10^−5^/ 3.28 × 10^−7^	no	0.55/0.53	6.4/7.1	Missense variant	Moderate	Protein plastid movement impaired Lipid phosphate phosphatase (LPP) in LD (~61.8 kbp)
	C18:3	2021/ 2022	contig 7845 77914	0.96	1.23 × 10^−6^/ 7.65 × 10^−6^	no	−0.6/−0.47	6/3.98	Synonymous variant	Low	Unknown
	C18:3	2022	chr1L 607096754	0.94	5.57 × 10^−6^	no	0.60	4.85	Intron variant	Modifier	Reticulon protein b21 1-acyl-sn-glycerol−3-phosphate acyltransferase (ATS2) in LD (~188 kbp)
	C18:3	2022	chr3 353865653	0.96	6.14 × 10^−6^	no	0.55	4.84	Intron variant	Modifier	3-oxoacyl-[acyl carrier protein]-synthase (KAS)
Lipid- derived	Hexanal	2021	chr1L 841732900	0.94	1.01 × 10^−6^	yes	−818	21.6	Missense variant	Moderate	Pentatricopeptide repeat containing protein
	1-Linoleoyl glycerol	2022	chr1L 1034384645	0.86	1.05 × 10^−5^	no	−0.12	22.77	Downstream gene variant	Modifier	Phosphopantetheine adenylyltransferase isoform
	2- Hydroxyoleic acid (OHOA)	2022	chr4 1463710346	0.78	7.78 × 10^−6^	no	1.51	19.13	Missense variant	Moderate	Rab gap tbc domain containing protein
Phenolic acid	p-coumaric acid	2021	chr2 964372547	0.94	7.81 × 10^−9^	yes	−9.54	27.8	Intron variant	Modifier	Quality protein dual specificity protein phosphatase phs Helix-loop-helix transcription factor (bHLH) 137-LIKE in LD (~450.5 kbp)
	p-coumaric acid	2022	chr1S 1040083079	0.93	1.40 × 10^−8^	yes	−0.04	27.23	Missense variant	Moderate	Protein kinase domain containing protein Transcription factor (MYB) in LD (~141.6 kbp)
	Caffeic acid	2021	chr4 6021087	0.96	2.75 × 10^−7^	yes	−2.11	28.2	Downstream gene variant	Modifier	oxygen evolving enhancer protein 3 1 chloroplastic Transcription factor (MYB) in LD (~120 kbp)
	Caffeic acid	2022	chr6 1188716500	0.95	7.98 × 10^−7^	yes	−0.61	24.92	Missense variant	Moderate	Shikimate kinase
Flavonoid	Quercetin Myricetin	2021	chr4 935741377	0.91	2.66 × 10^−5^ 1.68 × 10^−5^	no	−6.33 −16.5	16.7 17.4	Synonymous variant	Low	Beta amylase
Tannin	Catechin Epicatechin Procyanidin B1	2021/ 2022	chr2 671378841	0.94	8.7 × 10^−6^/ 1.9 × 10^−5^ 5.6 × 10^−6^/ 5.9 × 10^−6^ 3.8 × 10^−6^/ 2.6 × 10^−5^	no	14.4/2.5 11.9/3.4 22/4.7	8.9/8 10.4/8.5 10.1/7.1	Missense variant	Moderate	Unknown protein
	Epicatechin	2021/ 2022	chr2 826275103	0.92	5.06 × 10^−6^/ 1.96 × 10^−7^	no	13.7/3.7	13.29/14.9	Upstream gene variant	Modifier	Auxin responsive protein saur
	Procyanidin B1	2021/ 2022	chr2 953872991	0.89	1.22 × 10^−8^/ 2.75 × 10^−5^	no	21.4/3.5	17.9/8.4	Synonymous variant	Low	Calcium binding mitochondrial carrier protein scamc
	Procyanidin B1 Procyanidin B2	2022	chr2 953899416	0.93	3.14 × 10^−8^ 2.59 × 10^−5^	no	4.1 8.5	8.15 8.5	Intron variant	Modifier	Carbonyl reductase [nadph]
	Catechin Procyanidin B1 Procyanidin B2	2022	chr1L 672262213	0.95	1.65 × 10^−7^ 1.51 × 10^−7^ 3.6 × 10^−7^	yes	3.1 5.9 11.3	10.3 9.7 8.8	Intron variant	Modifier	Cathepsin
	Epicatechin	2022	chr2 1501542265	0.94	3.72 × 10^−7^	yes	3.58	7.27	Synonymous variant	Low	Cytochrome p450
Protein	Protein	2021	chr5 632641479	0.57	2.13 × 10^−5^	no	−0.87	10.7	Missense variant	Moderate	50s ribosomal protein l25 Cationic amino acid transporter 4 (CAT4) in LD (~136.8 kbp)

^a^ SNPs are identified with unique “chromosome position” codes (e.g., chr1S 1370573844). ^b^ Most of the reported SNPs are located within genes, and their effects are predicted (e.g., synonymous or missense variants). However, in addition to the genes harbouring these SNPs, we also report relevant candidate genes that are in linkage disequilibrium (“in LD”) and their distance from the SNPs.

## Data Availability

The datasets containing sequencing data generated and analysed during the current study are available in the Genome Sequence Archive (GCA) repository under the accession number CRA017806, https://ngdc.cncb.ac.cn/gsa/browse/CRA017806 (accessed on 15 November 2024).

## Supplementary Materials

The following supporting information can be downloaded at: https://www.mdpi.com/article/10.3390/plants14020193/s1, Figure S1: Distribution of marker missing data. Figure S2: Quantile-Quantile Plot (QQ) for protein content and off-flavours (2021). Figure S3: Quantile-Quantile Plot (QQ) for protein content and off-flavours (2022). Table S1: SNPs distribution and coverage along the six faba bean chromosomes. Table S2: Type-A additive genetic correlation and their approximated standard error estimated in 2021. Table S3: Type-A additive genetic correlation and their approximated standard error estimated in 2022. Table S4: GWAS-detected SNPs in 2021 and candidate genes. Table S5: GWAS-detected SNPs in 2022 and candidate genes. Table S6: Genomic inflation factors (λ) of the GWAS models. Table S7: Standards used for quantification. Table S8: Retention times and mass traces used for quantification.

## Appendix A. Field Trial Analysis: Adjusted Mean, Heritability, Type-B and Type-A Additive Genetic Correlations

Phenotypic (chemical) data were analysed using linear mixed models fitted with the ASReml 4.2-R package [68]. To estimate the narrow sense heritability (*h*^2^), a single-trait, single-environment analysis was performed using raw data based on the following generic model (A1):

***y*** = **1***μ* + ***X******β*** + ***Z*****_1_*****u*** + ***Z*****_2_*****r*****_(b)_** + ***Z*****_3_*****c*****_(b)_** + ***e*** (A1)

where ***y*** represents a vector of raw phenotypes, *μ* the overall mean, ***β*** the fixed effect coefficients for blocks, ***u*** the random genetic effects, with ***u*** ∼ *N*(**0**, *σ_g_*^2^***G***), ***r*_(b)_** the random rows effects within blocks, with ***r*****_(b)_** ∼ *N*(**0**, *σ_r_*^2^***I_r_***), ***c_(b)_*** the random columns effect within blocks, with ***c_(b)_*** ∼ *N*(**0**, *σ_c_*^2^***I_c_***), and ***e*** denotes the random residual errors with ***e***∼ *N*(**0**, ***Σ***), and ***Σ*** = *σ_e_*^2^(***AR1_r_***⊗***AR1_c_***). The sign ⊗ denotes the Kronecker product of rows (***AR1_r_***) and columns (***AR1_c_***), which both have an autoregressive structures that accounts for micro-environmental variations between residuals. ***X*** and ***Z*** are incidence matrices of their respective orders, ***I*** identity matrices of their respective orders, and ***G*** is the Genomic Relationship Matrix described earlier.

After fitting the models, residuals were evaluated for normality using diagnostic plots. A few outliers exceeding three times the standard deviation of the standardized residuals were excluded. Model (A1) was re-fitted to calculate the narrow sense heritability (*h*^2^) as follows: $h^{2}=1-\frac{\left[mean\left(PEVg\right)\right]}{\sigma_{g}^{2}}$ [70], where *mean(PEV_g_)* represent the average prediction error variance (PEV) for the genotypes, and *σ_g_*^2^ denotes the estimated additive genetic variance.

To estimate an adjusted mean for each genotype (Y_adj_), a similar model to (A1) was fitted, with ***u*** as a fixed genetic effect. These adjusted genotype means were then used in the GWAS.

The type-B additive genetic correlations [69] were estimated by fitting a single-trait, multi-environment trial (MET) model using raw data (combined 2021 and 2022):

***y*** = **1***μ* + *X***_1_*****t*** + ***X*****_2_*****β*****_(t)_** + ***Z*****_1_*****u*****_(t)_** + ***Z*****_2_*****r*****_(bt)_** + ***Z*****_3_*****c*****_(bt)_** +***e*** (A2)

where ***y*** represents a vector of raw phenotypes, ***μ*** is their overall mean across years, ***t*** is a vector of fixed year effect, ***β*_(t)_** is a vector of fixed blocks effects within the years, ***u_(t)_*** is a vector of random genetic effects within years, with ***u_(t)_*** ∼ *N*(**0**, *σ_g_*^2^***G***⊗***U*_0_**) with ***G*** described earlier, and ***U*_0_** being the 2 × 2 (years) variance-covariance (vcov) matrix with a heterogeneous structure (corgh) that allows for year-specific *σ_g_*^2^ and additive genetic correlations (ρ_type-B_) estimation. ***r*_(bt)_** is the vector of random effects for rows within blocks within years, with ***r*_(bt)_**∼ *N*(**0**, *σ_r_*^2^***D_r_***), ***c*****_(bt)_** is the vector of random effects for columns within blocks within years, with ***c*_(bt)_**∼ *N*(**0**, *σ_c_*^2^***D_c_***), and ***e*** is the vector of residual errors, with ***e*** ∼ *N*(**0**, *σ_e_*^2^***D_e_***) and with heterogeneity defined as ***Σ** = σ_e_*^2^(***AR1_r_***⊗***AR1_c_***). ***D_r_***, ***D_c_***, and ***D_e_*** are block diagonal matrices where each year level has its own *σ_r_*^2^, *σ_c_*^2^, and *σ_e_*^2^, respectively. All other terms were previously described. A value of ρ_type-B_ close to one (obtained from the matrix ***U*_0_**) suggests minimal genotype-by-year (GxY) interaction. The incremental Wald statistic was used to assess the significance of the year as a covariate in the model.

To estimate the type-A additive genetic correlations among traits [69], a bivariate, single-environment model was fitted for each pair of traits using raw data:

***y*** = **1***μ_m_* + ***X*****_1_*****t*** + ***X*****_2_*****β*****_(t)_** + ***Z*****_1_*****u*****_(t)_** + ***Z*****_2_*****r*****_(bt)_** + ***Z*****_3_*****c*****_(bt)_** + ***e*** (A3)

where ***y*** represents the stacked binary vector of phenotypes for two traits **y** = [**y_1_**, **y_2_**], and *μ_m_* is the bivariate version of intercept (*μ*). ***X*****_2_*****β*****_(t)_**, ***Z*****_2_*****r*****_(bt)_**, and ***Z*****_3_*****c*****_(bt)_** are stacked design effects defined as previously in the model (A2), but with “**t**” now denoting the trait, ***u*_(t)_** is a stacked vector of random genetic effects within trait, with ***u*_(t)_** ∼ *N*(**0**, *σ_g_*^2^***G***⊗***U*****_0_**), ***G*** is previously described, ***U*****_0_** is a 2 × 2 (traits) vcov matrix that is unstructured (corgh), allowing for trait-specific *σ_g_*^2^ and unique additive genetic correlations across traits (ρ_type-A_), ***e*** is a stacked vector of random residual effects, with ***e*** ∼ *N*(**0**, *σ_e_*^2^***I***⊗***R*****_0_**), and ***R*****_0_** is the vcov between errors that is heterogeneous (corgh), introducing residual correlations between traits and unequal *σ_e_*^2^ across traits. To aid convergence in REML for the bivariate model, the starting values for *σ_g_*^2^ and *σ_e_*^2^ were derived from the univariate model (A1) for each trait.

Standard errors (approximated) for ρ_type-B_ and ρ_type-A_ correlations were estimated using the Delta method [71].

For phenolic acids, flavonoids, and lipid-derived products, models (A1), (A2) and (A3) were simplified by excluding design terms ***Xβ***, ***Z*****_2_*****r*****_(b)_**, and ***Z*****_3_*****c*****_(b)_** (block, row within block and column within block). In fact, NIRS prediction failure for these traits led to missing values and partially replicated data, preventing the robust estimation of block, row and column effects.
