# Supplementary material for: GWAS Identifies SNP Markers and Candidate Genes for Off-Flavours and Protein Content in Faba Bean (Vicia faba L.)
Source: Plants (Basel). 2025 Jan 11;14(2):193. doi: 10.3390/plants14020193 (PMC11768279; doi:10.3390/plants14020193)
Supplement: Supplementary file 1 [file plants-14-00193-s001.zip › Figures S1-S3.pdf]

GWAS identifies SNP markers and candidate genes for off-flavors and seed quality in faba bean (*Vicia Faba* L.)

Antonio Lippolis[1], Boudewijn Hollebrands[2,3], Valentina Acierno[4], Catrienus de Jong[4], Laurice Pouvreau[4], João Paulo[5], Salvador A Gezan[5], Luisa M Trindade[1]

[1] Plant Breeding, Wageningen University & Research, Droevendaalsesteeg 1, 6708PB, Wageningen, the Netherlands

[2] Unilever Foods Innovation Centre – Hive, Bronland 14, 6708 WH, Wageningen, the Netherlands

[3] Laboratory of Organic Chemistry, Wageningen University & Research, Stippeneng 4, 6708 WE, Wageningen, the Netherlands

[4] Wageningen Food & Biobased Research, Wageningen University & Research, Bornse Weilanden 9, 6708WG, Wageningen, The Netherlands

[5] Biometris, Wageningen University & Research, Droevendaalsesteeg 1, 6708PB, Wageningen, the Netherlands

[6] VSN International Ltd, Hemel Hempstead, HP2 4TP, United Kingdom

Email: **AL** antonio.lippolis@wur.nl; **BH** Boudewijn.Hollebrands@unilever.com; **VA** valentina.acierno@wur.nl; **CdJ** catrienus.dejong@wur.nl; **LP** laurice.povreau@wur.nl; **JP** joao.paulo@wur.nl; **SG** salvador.gezan@vsni.co.uk; **LMT** luisa.trindade@wur.nl

This supplementary material (figures) file totalling four pages contains three supplementary figures, Supplementary Figures S1-S3.

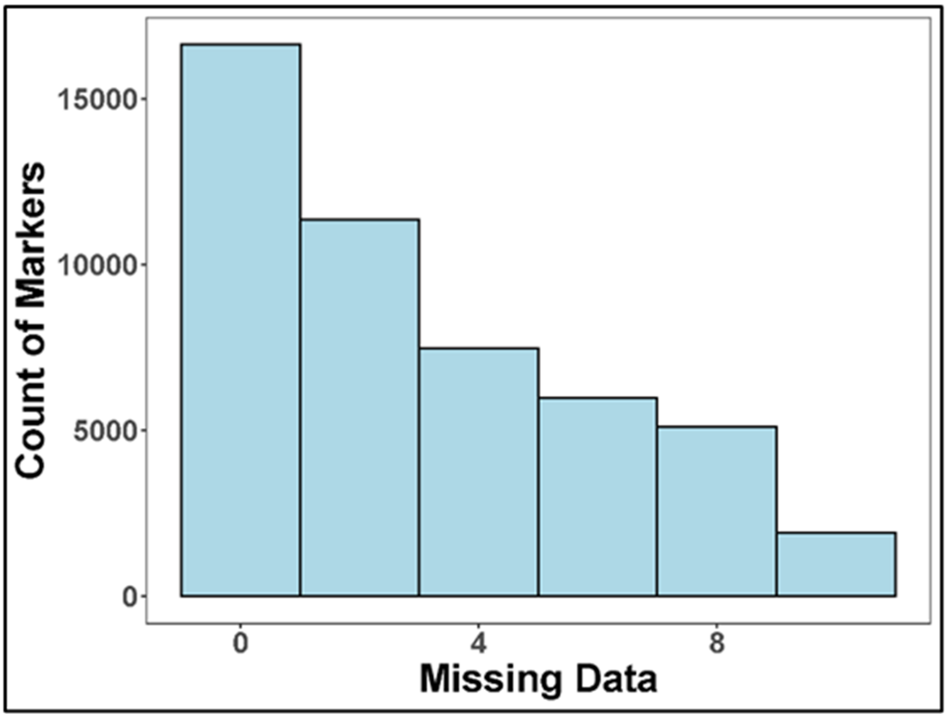

Supplementary Figure S1: Distribution of marker missing data. The maximum allowed missing rate was 10%. A total of 73% of the markers had missing rate between 0% and 5%.

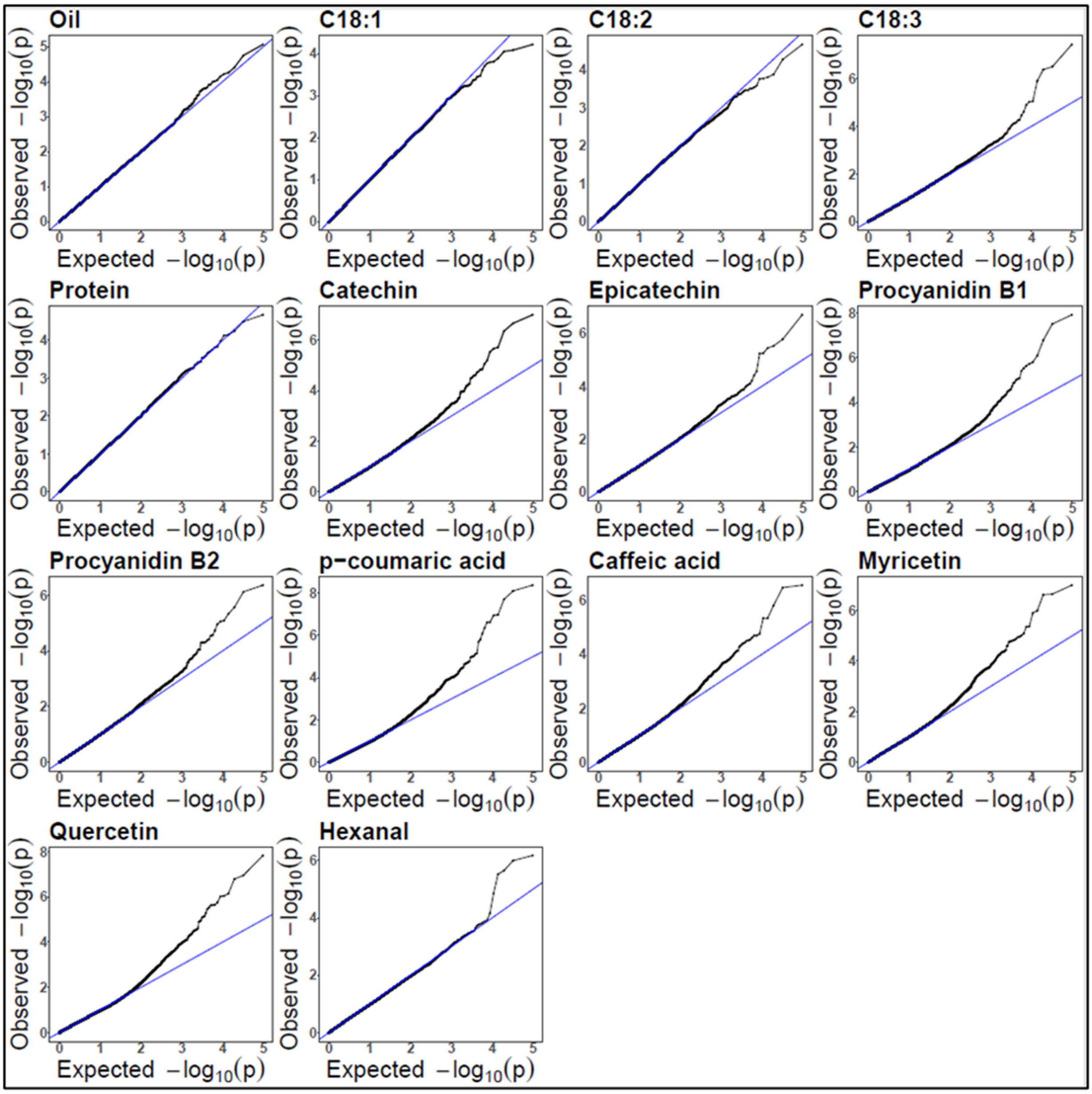

Supplementary Figure S2: Quantile-Quantile Plot (QQ) for protein content and off-flavours (2021). The x-axis represents the expected $-\log_{10}$(p-value under the null hypothesis, and the y-axis shows the observed $-\log_{10}$(p-value) from the GWAS results. Points along the diagonal indicate conformity to the null hypothesis, while deviations suggest SNP associations.

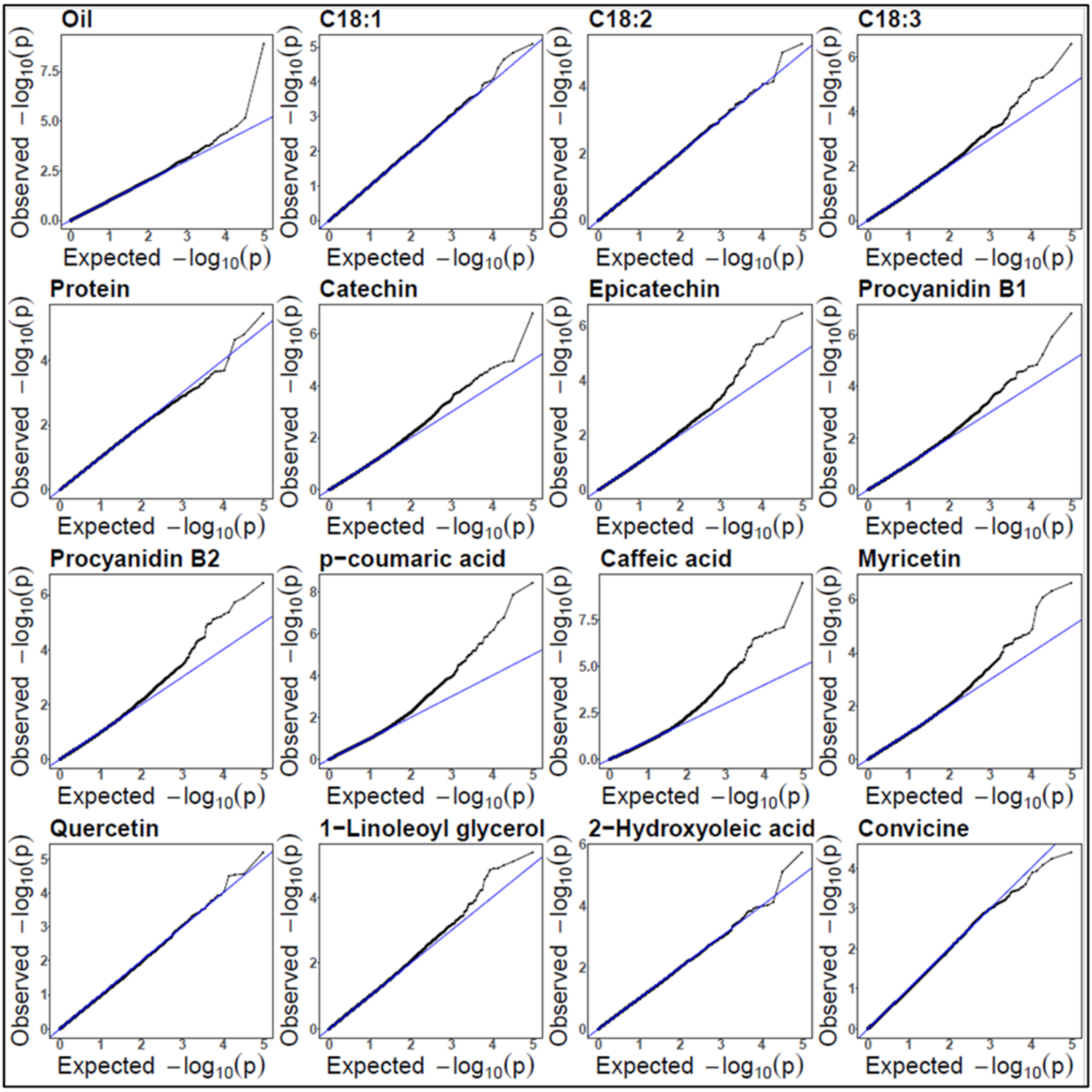

Supplementary Figure S3: Quantile-Quantile Plot (QQ) for protein content and off-flavours (2022). The x-axis represents the expected $-\log_{10}$(p-value under the null hypothesis, and the y-axis shows the observed $-\log_{10}$(p-value) from the GWAS results. Points along the diagonal indicate conformity to the null hypothesis, while deviations suggest SNP associations.
