# Supplementary material for: GWAS Identifies SNP Markers and Candidate Genes for Off-Flavours and Protein Content in Faba Bean (Vicia faba L.)
Source: Plants (Basel). 2025 Jan 11;14(2):193. doi: 10.3390/plants14020193 (PMC11768279; doi:10.3390/plants14020193)
Supplement: Supplementary file 1 [file plants-14-00193-s001.zip › Table S1-S3 and S6.pdf]

# GWAS identifies SNP markers and candidate genes for off-flavors and seed quality in faba bean (*Vicia Faba* L.)

Antonio Lippolis[1], Boudewijn Hollebrands[2,3], Valentina Acierno[4], Catrienus de Jong[4], Laurice Pouvreau[4], João Paulo[5], Salvador A Gezan[5], Luisa M Trindade[1]

[1] Plant Breeding, Wageningen University & Research, Droevendaalsesteeg 1, 6708PB, Wageningen, the Netherlands

[2] Unilever Foods Innovation Centre – Hive, Bronland 14, 6708 WH, Wageningen, the Netherlands

[3] Laboratory of Organic Chemistry, Wageningen University & Research, Stippeneng 4, 6708 WE, Wageningen, the Netherlands

[4] Wageningen Food & Biobased Research, Wageningen University & Research, Bornse Weilanden 9, 6708WG, Wageningen, The Netherlands

[5] Biometris, Wageningen University & Research, Droevendaalsesteeg 1, 6708PB, Wageningen, the Netherlands

[6] VSN International Ltd, Hemel Hempstead, HP2 4TP, United Kingdom

Email: **AL** antonio.lippolis@wur.nl; **BH** Boudewijn.Hollebrands@unilever.com; **VA** valentina.acierno@wur.nl; **CdJ** catrienus.dejong@wur.nl; **LP** laurice.povreau@wur.nl; **JP** joao.paulo@wur.nl; **SG** salvador.gezan@vsni.co.uk; **LMT** luisa.trindade@wur.nl

This supplementary material (tables) file totalling five pages contains four supplementary tables, Supplementary Tables S1, S2, S3 and S6. Please note that Supplementary Tables S4 and S5 are provided in other file.

**Supplementary Table S1:** SNPs distribution and coverage along the six faba bean chromosomes.

| Chromosome | Total SNPs [a] | Covered distance (Mbp)[b] | Average density (SNPs/Mbp) | Max gap (Mbp)[c] |
|---|---|---|---|---|
| chr1L | 8050 | 1801,79 | 4,47 | 31,2 |
| chr1S | 5176 | 1568,29 | 3,3 | 28,31 |
| chr2 | 7783 | 1716,59 | 4,53 | 22,08 |
| chr3 | 7172 | 1637,24 | 4,38 | 44,14 |
| chr4 | 6285 | 1643,87 | 3,82 | 60,05 |
| chr5 | 5600 | 1364,87 | 4,1 | 69,9 |
| chr6 | 5783 | 1519,9 | 3,8 | 85,56 |
| conting | 2596 | - | - | - |

[a] 48,445 SNPs selected for GWAS.

[b] physical distance between the first and last SNP marker on the same chromosome in Megabase pair (Mbp).

[c] maximum physical distance existing between two neighbouring SNP markers in Megabase pair (Mbp).

**Supplementary Table S2**: Type-A additive genetic correlation and their approximated standard error estimated in 2021.

| | Oil | C18:1 | C18:2 | C18:3 | Protein | Catechin | Epicatechin | Procyanidin B1 | Procyanidin B2 | Hexanal | p-coumaric acid | Caffeic acid | Myricetin | Quercetin |
|---|---|---|---|---|---|---|---|---|---|---|---|---|---|---|
| **Oil** | 1 | 0.77 (0.05) | -0.48 (0.09) | -0.76 (0.05) | -0.28 (0.14) | 0.29 (0.1) | -0.02 (0.1) | 0.19 (0.1) | 0.07 (0.1) | -0.09 (0.17) | -0.09 (0.14) | 0.07 (0.14) | 0.08 (0.14) | -0.08 (0.13) |
| **C18:1** | 0.77 (0.05) | 1 | -0.68 (0.06) | -0.87 (0.03) | -0.19 (0.12) | 0.54 (0.08) | 0.23 (0.09) | 0.45 (0.08) | 0.25 (0.09) | -0.29 (0.15) | 0 (0.13) | 0.12 (0.12) | 0.21 (0.13) | 0.17 (0.12) |
| **C18:2** | -0.48 (0.09) | -0.68 (0.06) | 1 | 0.36 (0.1) | 0.37 (0.13) | -0.27 (0.11) | -0.14 (0.11) | -0.29 (0.1) | -0.13 (0.11) | 0.39 (0.17) | -0.04 (0.15) | -0.13 (0.15) | 0.01 (0.15) | 0.01 (0.13) |
| **C18:3** | -0.76 (0.05) | -0.87 (0.03) | 0.36 (0.1) | 1 | 0.25 (0.12) | -0.55 (0.07) | -0.2 (0.09) | -0.44 (0.08) | -0.23 (0.09) | 0.1 (0.16) | -0.01 (0.13) | -0.09 (0.12) | -0.05 (0.13) | -0.01 (0.12) |
| **Protein** | -0.28 (0.14) | -0.19 (0.12) | 0.37 (0.13) | 0.25 (0.12) | 1 | -0.14 (0.14) | -0.16 (0.13) | -0.24 (0.12) | 0.02 (0.13) | -0.29 (0.19) | -0.07 (0.18) | -0.12 (0.17) | 0.12 (0.17) | 0.14 (0.16) |
| **Catechin** | 0.29 (0.1) | 0.54 (0.08) | -0.27 (0.11) | -0.55 (0.07) | -0.14 (0.14) | 1 | 0.65 (0.06) | 0.92 (0.02) | 0.66 (0.06) | -0.37 (0.15) | -0.2 (0.13) | -0.05 (0.13) | 0.21 (0.13) | 0.15 (0.12) |
| **Epicatechin** | -0.02 (0.1) | 0.23 (0.09) | -0.14 (0.11) | -0.2 (0.09) | -0.16 (0.13) | 0.65 (0.06) | 1 | 0.84 (0.03) | 0.92 (0.02) | -0.2 (0.15) | 0.1 (0.12) | 0.29 (0.11) | 0.24 (0.12) | 0.2 (0.11) |
| **Procyanidin B1** | 0.19 (0.1) | 0.45 (0.08) | -0.29 (0.1) | -0.44 (0.08) | -0.24 (0.12) | 0.92 (0.02) | 0.84 (0.03) | 1 | 0.8 (0.04) | -0.36 (0.14) | -0.06 (0.12) | 0.15 (0.12) | 0.2 (0.11) | 0.12 (0.11) |
| **Procyanidin B2** | 0.07 (0.1) | 0.25 (0.09) | -0.13 (0.11) | -0.23 (0.09) | 0.02 (0.13) | 0.66 (0.06) | 0.92 (0.02) | 0.8 (0.04) | 1 | -0.25 (0.14) | 0.07 (0.12) | 0.29 (0.11) | 0.25 (0.12) | 0.25 (0.11) |
| **Hexanal** | -0.09 (0.17) | -0.29 (0.15) | 0.39 (0.17) | 0.1 (0.16) | -0.29 (0.19) | -0.37 (0.15) | -0.2 (0.15) | -0.36 (0.14) | -0.25 (0.14) | 1 | 0.05 (0.17) | -0.07 (0.16) | -0.05 (0.17) | -0.27 (0.15) |
| **p-coumaric acid** | -0.09 (0.14) | 0 (0.13) | -0.04 (0.15) | -0.01 (0.13) | -0.07 (0.18) | -0.2 (0.13) | 0.1 (0.12) | -0.06 (0.12) | 0.07 (0.12) | 0.05 (0.17) | 1 | 0.36 (0.11) | -0.13 (0.14) | 0.08 (0.12) |
| **Caffeic acid** | 0.07 (0.14) | 0.12 (0.12) | -0.13 (0.15) | -0.09 (0.12) | -0.12 (0.17) | -0.05 (0.13) | 0.29 (0.11) | 0.15 (0.12) | 0.29 (0.11) | -0.07 (0.16) | 0.36 (0.11) | 1 | 0.09 (0.14) | 0.04 (0.12) |
| **Myricetin** | 0.08 (0.14) | 0.21 (0.13) | 0.01 (0.15) | -0.05 (0.13) | 0.12 (0.17) | 0.21 (0.13) | 0.24 (0.12) | 0.2 (0.11) | 0.25 (0.12) | -0.05 (0.17) | -0.13 (0.14) | 0.09 (0.14) | 1 | 0.71 (0.14) |
| **Quercetin** | -0.08 (0.13) | 0.17 (0.12) | 0.01 (0.13) | -0.01 (0.12) | 0.14 (0.16) | 0.15 (0.12) | 0.2 (0.11) | 0.12 (0.11) | 0.25 (0.11) | -0.27 (0.15) | 0.08 (0.12) | 0.04 (0.12) | 0.71 (0.14) | 1 |

**Supplementary Table S3:** Type-A additive genetic correlation and their approximated standard error estimated in 2022.

| | Oil | C18:1 | C18:2 | C18:3 | Protein | Catechin | Epicatechin | Procyanidin B1 | Procyanidin B2 | p-coumaric acid | Caffeic acid | Myricetin | Quercetin | 1-Linoleoyl glycerol | OHOA | Convicine |
|---|---|---|---|---|---|---|---|---|---|---|---|---|---|---|---|---|
| **Oil** | 1 | 0.74 (0.04) | -0.35 (0.09) | -0.66 (0.05) | -0.22 (0.09) | -0.09 (0.09) | 0.04 (0.08) | -0.03 (0.09) | -0.04 (0.09) | 0.21 (0.15) | -0.1 (0.14) | 0.13 (0.12) | 0.32 (0.26) | 0.05 (0.2) | 0.21 (0.15) | 0.02 (0.14) |
| **C18:1** | 0.74 (0.04) | 1 | -0.47 (0.07) | -0.8 (0.03) | -0.1 (0.1) | 0.08 (0.09) | 0.3 (0.08) | 0.16 (0.08) | 0.19 (0.08) | 0.17 (0.14) | -0.05 (0.14) | 0.11 (0.12) | 0.38 (0.22) | -0.05 (0.19) | 0.12 (0.14) | -0.31 (0.13) |
| **C18:2** | -0.35 (0.09) | -0.47 (0.07) | 1 | 0.06 (0.1) | 0.39 (0.09) | -0.27 (0.09) | -0.34 (0.08) | -0.4 (0.08) | -0.39 (0.08) | -0.15 (0.14) | 0.12 (0.14) | -0.07 (0.12) | -0.57 (0.38) | 0.07 (0.2) | -0.05 (0.15) | 0.24 (0.15) |
| **C18:3** | -0.66 (0.05) | -0.8 (0.03) | 0.06 (0.1) | 1 | 0.21 (0.09) | -0.15 (0.08) | -0.24 (0.08) | -0.18 (0.08) | -0.19 (0.08) | -0.03 (0.14) | -0.09 (0.14) | 0.02 (0.12) | -0.17 (0.21) | 0.18 (0.17) | 0.04 (0.14) | 0.25 (0.13) |
| **Protein** | -0.22 (0.09) | 0.1 | 0.39 (0.09) | 0.21 (0.09) | 1 | -0.51 (0.07) | -0.46 (0.07) | -0.56 (0.06) | -0.46 (0.07) | -0.13 (0.16) | -0.23 (0.15) | -0.18 (0.13) | -0.75 (0.36) | 0.12 (0.2) | 0.29 (0.16) | 0.19 (0.15) |
| **Catechin** | -0.09 (0.09) | 0.08 (0.09) | -0.27 (0.09) | -0.15 (0.08) | -0.51 (0.07) | 1 | 0.69 (0.04) | 0.94 (0.01) | 0.93 (0.01) | -0.22 (0.12) | 0.02 (0.13) | 0.14 (0.11) | 0.5 (0.22) | -0.35 (0.15) | -0.15 (0.13) | -0.28 (0.12) |
| **Epicatechin** | 0.04 (0.08) | 0.3 (0.08) | -0.34 (0.08) | -0.24 (0.08) | -0.46 (0.07) | 0.69 (0.04) | 1 | 0.77 (0.03) | 0.83 (0.02) | -0.1 (0.11) | 0.21 (0.11) | 0.18 (0.1) | 0.72 (0.36) | -0.16 (0.15) | -0.12 (0.12) | -0.3 (0.11) |
| **Procyanidin B1** | -0.03 (0.09) | 0.16 (0.08) | -0.4 (0.08) | -0.18 (0.08) | -0.56 (0.06) | 0.94 (0.01) | 0.77 (0.03) | 1 | 0.98 (0) | -0.17 (0.12) | 0.03 (0.12) | 0.21 (0.1) | 0.62 (0.29) | -0.32 (0.16) | -0.14 (0.12) | -0.3 (0.12) |
| **Procyanidin B2** | -0.04 (0.09) | 0.19 (0.08) | -0.39 (0.08) | -0.19 (0.08) | -0.46 (0.07) | 0.93 (0.01) | 0.83 (0.02) | 0.98 (0.02) | 1 | -0.2 (0.12) | 0.03 (0.12) | 0.2 (0.1) | 0.59 (0.28) | -0.35 (0.16) | -0.12 (0.12) | -0.37 (0.12) |
| **p-coumaric acid** | 0.21 (0.15) | 0.17 (0.14) | -0.15 (0.14) | -0.03 (0.14) | -0.13 (0.16) | -0.22 (0.12) | -0.1 (0.11) | -0.17 (0.12) | -0.2 (0.12) | 1 | 0.29 (0.17) | 0.07 (0.13) | 0.54 (0.33) | -0.16 (0.24) | -0.37 (0.16) | -0.02 (0.15) |
| **Caffeic acid** | -0.1 (0.14) | -0.05 (0.14) | 0.12 (0.14) | -0.09 (0.14) | -0.23 (0.15) | 0.02 (0.13) | 0.21 (0.11) | 0.03 (0.12) | 0.03 (0.12) | 0.29 (0.17) | 1 | 0.04 (0.12) | 0.23 (0.24) | 0.07 (0.25) | -0.1 (0.15) | -0.09 (0.14) |
| **Myricetin** | 0.13 (0.12) | 0.11 (0.12) | -0.07 (0.12) | 0.02 (0.12) | -0.18 (0.13) | 0.14 (0.11) | 0.18 (0.1) | 0.21 (0.1) | 0.2 (0.1) | 0.07 (0.13) | 0.04 (0.12) | 1 | 0.32 (0.23) | 0.05 (0.17) | -0.09 (0.13) | -0.35 (0.12) |
| **Quercetin** | 0.32 (0.26) | 0.38 (0.22) | -0.57 (0.38) | -0.17 (0.21) | -0.75 (0.36) | 0.5 (0.22) | 0.72 (0.36) | 0.62 (0.29) | 0.59 (0.28) | 0.54 (0.33) | 0.23 (0.24) | 0.32 (0.23) | 1 | 0.15 (0.41) | -0.13 (0.28) | -0.18 (0.29) |
| **1-linoleoyl glycerol** | 0.05 (0.2) | -0.05 (0.19) | 0.07 (0.2) | 0.18 (0.17) | 0.12 (0.2) | -0.35 (0.15) | -0.16 (0.15) | -0.32 (0.16) | -0.35 (0.16) | -0.16 (0.24) | 0.07 (0.25) | 0.05 (0.17) | 0.15 (0.41) | 1 | 0.75 (0.15) | 0.46 (0.19) |
| **OHOA** | 0.21 (0.15) | 0.12 (0.14) | -0.05 (0.15) | 0.04 (0.14) | 0.29 (0.16) | -0.15 (0.13) | -0.12 (0.12) | -0.14 (0.12) | -0.12 (0.12) | -0.37 (0.16) | -0.1 (0.15) | -0.09 (0.13) | -0.13 (0.28) | 0.75 (0.15) | 1 | 0.47 (0.12) |
| **Convicine** | 0.02 (0.14) | -0.31 (0.13) | 0.24 (0.15) | 0.25 (0.13) | 0.19 (0.15) | -0.28 (0.12) | -0.3 (0.11) | -0.3 (0.12) | -0.37 (0.12) | -0.02 (0.15) | -0.09 (0.14) | -0.35 (0.12) | -0.18 (0.29) | 0.46 (0.19) | 0.47 (0.12) | 1 |

**Supplementary Table S6:** Genomic inflation factors (λ) of the GWAS models.

| Trait | 2021 | 2022 |
|---|---|---|
| Oil | 0.99 | 1.01 |
| C18:1 | 0.97 | 0.99 |
| C18:2 | 1.0 | 0.99 |
| C18:3 | 0.95 | 0.97 |
| Protein | 1.02 | 1.01 |
| Catechin | 0.94 | 0.96 |
| Epicatechin | 0.97 | 0.98 |
| Procyanidin B1 | 0.93 | 0.94 |
| Procyanidin B2 | 0.95 | 0.94 |
| p-coumaric acid | 0.95 | 1.03 |
| Caffeic acid | 1.0 | 0.9 |
| Myricetin | 1.05 | 1.0 |
| Quercetin | 1.0 | 0.96 |
| 1-Linoleoyl glycerol | - | 1.04 |
| 2-Hydroxyoleic acid (OHOA) | - | 1.01 |
| Convicine | - | 0.95 |
| Hexanal | 1.0 | - |
