# Supplementary material for: GWAS Identifies SNP Markers and Candidate Genes for Off-Flavours and Protein Content in Faba Bean (Vicia faba L.)
Source: Plants (Basel). 2025 Jan 11;14(2):193. doi: 10.3390/plants14020193 (PMC11768279; doi:10.3390/plants14020193)
Supplement: Supplementary file 1 [file plants-14-00193-s001.zip › Tables S7 and S8.pdf]

GWAS identifies SNP markers and candidate genes for off-flavors and seed quality in faba bean (*Vicia Faba* L.)

Antonio Lippolis[1], Boudewijn Hollebrands[2,3], Valentina Acierno[4], Catrienus de Jong[4], Laurice Pouvreau[4], João Paulo[5], Salvador A Gezan[5], Luisa M Trindade[1]

[1] Plant Breeding, Wageningen University & Research, Droevendaalsesteeg 1, 6708PB, Wageningen, the Netherlands

[2] Unilever Foods Innovation Centre – Hive, Bronland 14, 6708 WH, Wageningen, the Netherlands

[3] Laboratory of Organic Chemistry, Wageningen University & Research, Stippeneng 4, 6708 WE, Wageningen, the Netherlands

[4] Wageningen Food & Biobased Research, Wageningen University & Research, Bornse Weilanden 9, 6708WG, Wageningen, The Netherlands

[5] Biometris, Wageningen University & Research, Droevendaalsesteeg 1, 6708PB, Wageningen, the Netherlands

[6] VSN International Ltd, Hemel Hempstead, HP2 4TP, United Kingdom

Email: **AL** antonio.lippolis@wur.nl; **BH** Boudewijn.Hollebrands@unilever.com; **VA** valentina.acierno@wur.nl; **CdJ** catrienus.dejong@wur.nl; **LP** laurice.povreau@wur.nl; **JP** joao.paulo@wur.nl; **SG** salvador.gezan@vsni.co.uk; **LMT** luisa.trindade@wur.nl

This Supplementary Methods file totaling five pages contains a detailed protocol on the LC-MS profiling in faba bean extracts, complementing the "4.2.5 Liquid Chromatography-Mass Spectrometry (LC-MS) of non VOCs" paragraph of the main manuscript.

Supplementary Methods

LC-MS Profiling in Faba bean extracts

Materials

Acetonitrile, methanol, formic acid (ULC/MS grade) were obtained from Biosolve (Valkenswaard, The Netherlands).

Standards

Stock solutions of numerous standards were prepared. 1 mL of each of the standard solutions was accurately pippeted to prepare a stock solution mix. The stock solution was diluted to create calibration lines by dilution with a 1:1 (v/v) mixture of methanol and water acidified with 1% formic acid, according to Table 1.

Sample preparation

The grinded faba bean material was extracted by applying solid liquid extraction. 500 mg of each sample was accurately weighed into separate 15 mL Falcon tubes. To each tube, 10 mL of an extraction solvent consisting of a 1:1 (v/v) mixture of methanol and water, acidified with 1% formic acid, was added. The samples were then subjected to ultrasonication for 1 hour with occasional manual shaking. After sonication, the samples were centrifuged at 3000 × g for 15 minutes. From each sample extract, 1 mL of the supernatant was pipetted into individual Eppendorf tubes of 1.5 mL and an additional centrifugation step was applied using a table centrifuge (VWR Microstar 17R) for 12 min at 13.300x rpm under constant cooling at 4 °C. The resulting clear supernatant was transferred into HPLC vials for subsequent analysis by LC-MS.

Preparation QC sample

Of each sample extract 100 µL was pippeted into a 50 mL Falcon tube, this pooled aliquot was used during the study as QC-sample.

LC-MS Method description

All LC-HRMS analyses were conducted using an UltiMate 3000 RS chromatography system, connected to a Q-Exactive Plus Hybrid Quadrupole-Orbitrap mass spectrometer (Thermo Fischer Scientific, Waltham MA, USA).

Separations were performed using a BEH C18 column (2.1 mm x 100 mm, 1.7 µm; Waters, Etten-Leur, The Netherlands) with associated VanGuard pre-column. The analytes were eluted at a flow rate of 0.30 mL/min using a linear gradient of water (solvent A) and acetonitrile (solvent B), both containing 0.1% formic acid. The gradient was programmed as follows: 1 min at 0.1% B, ramping to 40% B in 11.5 min, then to 100%B in 2.5 min, maintained at 100%B for 2.5 min, followed by return to 0.1% B over 0.1 minute, and finally a 4.4-minute re-equilibration at 0.1% B. The total run time was 22 minutes. The column temperature was maintained at 40 °C and the autosampler was kept at 5 °C. The injection volume was 5 µL.

The electrospray ion source was operated in both positive and negative mode with the following parameters: the capillary temperature was set to 320 °C, the heater temperature was 350 °C, the sheath gas to 50 arbitrary units, and the auxiliary gas to 13 arbitrary units. The capillary voltage was set to 3.3 kV with an S-lens RF level of 50. Calibration was achieved using Pierce Positive and Negative Ion Calibration Solution (Thermo Fischer). Data acquisition was carried out using Xcalibur 3.1.

For LC-MS profiling the mass spectrometer was operated in full-scan MS mode. Spectra were acquired in both positive and negative ionization mode at a resolution of 70000 over an *m/z* range of 100–1500, with a maximum ion injection time of 100 milliseconds.

For identification analysis were performed in full-scan data-dependent MS2 (ddMS2) mode on a pooled sample. ddMS2 scans were acquired at a mass resolution of 17500, with an isolation window of 2 m/z, of the top 10 most abundant ions selected from MS survey scans acquired at a mass resolution of 70000. The maximum injection time of the ddMS2 scans was set to 50 milliseconds and a normalized stepped collision energy was applied of 15, 30 and 60 arbitrary units.

Sample sequence

The complete set of sample extracts was measured by LC-MS profiling over multiple sessions. In each session, analytical blanks (extraction solvent), standard dilutions, sample extracts and the pooled QC-sample were analyzed. Each sample extract was measured in triplicate and QC samples were measured after every ten LC-MS analyses. In each sessions identification by ddMS2 analysis was performed on the QC-sample.

LC-MS targeted data

Quantification was conducted using Xcalibur 3.1 (Thermo). External calibration lines were established for each standard using the integrated peak response values of the standard dilutions shown in Table 1. The integrated peak response was determined by generation of extracted ion chromatograms for each compound using either the accurate m/z value of the [M+H] or [M-H] ion with a mass window of 5 ppm. The calibration lines and the integrated peak response were used to calculate the measured concentration in solution. In Table 2 the retention times and the m/z of the ions used for quantification are listed.

Supplementary Methods Table S7: Standards used for quantification.

Stock solution		Concentration (ug/L) Mix	10x dil	L1	L2	L3	L4	L5	L6
Caffeic acid	18.0 mg/100ml 75% MeOH	6670	667	222	111	56	37	22	11
p-Coumaric acid	12.4 mg/100ml 75% MeOH	4590	459	153	77	31	20	15	8
Ferulic acid	19.6 mg/100ml 75% MeOH	7260	726	242	121	48	32	24	12
Myricetin	15.6 mg/100ml MeOH	5780	578	193	96	39	26	19	10
Quercetin	19.6 mg/100ml MeOH	7260	726	242	121	48	32	24	12
(+)-Catechin	8.4 mg/100ml 75% MeOH	3110	311	104	52	26	17	10	5
(-)-Epicatechin	12.0 mg/100ml 75% MeOH	4440	444	148	74	37	25	15	7
Procyanidin B1	20 mg/100ml MeOH	7410	741	247	124	49	33	25	12
Procyanidin B2	20 mg/100ml MeOH	7410	741	247	124	49	33	25	12
1-Linoleoyl-glycerol (C18:1)	100 mg/50 ml MeOH	66667	6667	2222	1111	444	296	222	111
Convicine	1 mg/5 ml MeOH	6667	667	222	111	44	30	22	11
Linolenic acid (C18:3)	100 mg/100 ml MeOH	33333	3333	1111	556	222	148	111	56
2-Hydroxyoleic acid (2-OHOA)	5 mg/10 ml MeOH	16667	1667	556	278	139	93	56	28

Supplementary Methods Table S8: Retention times and mass traces used for quantification.

	LCMS rt (min)	Polarity
Catechin	7,04	Neg
Epicatechin	7,82	Neg
Procyanidin B1	6,76	Neg
Procyanidin B2	7,03	Neg
p-Coumaric acid	9,11	pos
Caffeic acid	7,71	pos
Ferulic acid	9,6	pos
Myricetin	9,42	pos
Quercetin	12,64	pos
1-Linoleoyl glycerol	17,18	pos
2-Hydroxyoleic acid (OHOA)	17,76	pos
Convicine	1,7	pos
